# Early brain radiotherapy combined with third-generation EGFR-TKIs improves survival in EGFR-mutant NSCLC with synchronous brain metastases: a multi-center retrospective analysis

**DOI:** 10.3389/fonc.2026.1770066

**Published:** 2026-02-12

**Authors:** Yaru Kong, Yunchuan Sun, Li Xiao, Hongling Lu, Rui Wang, Yongchao Yu, Jianxi Zhou, Jiaju Zhang, Yingnan Zhou

**Affiliations:** 1 Department of Head, Neck and Thoracic Oncology, Cangzhou Hospital of Integrated Traditional Chinese and Western Medicine-Hebei, Cangzhou, Hebei, China; 2 Key Laboratory of Cancer Prevention and Therapy, Tianjin Medical University Cancer Institute and Hospital, National Clinical Research Center for Cancer, Tianjin, China; 3 Tianjin’s Clinical Research Center for Cancer, Department of Thoracic Oncology, Tianjin Cancer Institute & Hospital, Tianjin Medical University, Tianjin, China; 4 Tianjin Lung Cancer Center, Department of Thoracic Oncology, Tianjin Cancer Institute & Hospital, Tianjin Medical University, Tianjin, China; 5 Department of Hepatobiliary Surgery I, Cangzhou Hospital of Integrated Traditional Chinese and Western Medicine-Hebei, Cangzhou, Hebei, China; 6 Department of Radiotherapy and Chemotherapy, East Branch, Cangzhou Hospital of Integrated Traditional Chinese and Western Medicine-Hebei, Cangzhou, Hebei, China

**Keywords:** brain metastases, EGFR mutation, hippocampal-avoidance radiotherapy, non-small cell lung cancer, stereotacticradiosurgery, treatment sequencing

## Abstract

**Objective:**

The optimal timing for combining brain radiotherapy with first-line third-generation EGFR tyrosine kinase inhibitors (TKIs) in patients with EGFR-mutant non-small cell lung cancer (NSCLC) and synchronous brain metastases (BM) remains uncertain. We compared an early combined therapy (ECT) strategy with a salvage radiotherapy (SRT) strategy.

**Methodology:**

In this multi-center retrospective study, patients with newly diagnosed EGFR-mutant NSCLC and synchronous BM receiving first-line third-generation EGFR-TKIs were classified into ECT (radiotherapy within 90 days of TKI initiation without progression, n=83), SRT (radiotherapy at intracranial progression, n=83), and TKI monotherapy (n=27) groups. The primary endpoint was intracranial progression-free survival (iPFS). Secondary endpoints included overall survival (OS) and safety.

**Results:**

The ECT strategy significantly prolonged iPFS compared to SRT (median 22.4 vs. 15.7 months; hazard ratio [HR] 0.628, 95% CI 0.459–0.858; P = 0.002). OS was also significantly longer with ECT (median 37.5 vs. 31.8 months; HR 0.637, 95% CI 0.465–0.871; P = 0.003). The survival benefit was most pronounced in patients with 1–3 BMs and exon 19 deletions. Multivariate analysis confirmed ECT as an independent favorable prognostic factor. The incidence of grade ≥3 adverse events and specific neurotoxicities was low and comparable between groups.

**Conclusion:**

For patients with EGFR-mutant NSCLC and synchronous BM, early brain radiotherapy combined with first-line third-generation EGFR-TKIs is associated with significantly improved intracranial control and overall survival compared to deferring radiotherapy until progression, without a significant increase in severe toxicity. These findings support consideration of an early integrated treatment approach.

## 1 Introduction

Lung cancer remains the leading cause of cancer-related mortality globally, with non-small cell lung cancer (NSCLC) representing the predominant histological subtype (1). Brain metastases (BM) develop in approximately 44% of patients with advanced NSCLC, conferring a dismal prognosis and significantly impairing quality of life (2). The presence of activating mutations in the epidermal growth factor receptor (EGFR) gene, particularly exon 19 deletions and L858R point mutations, is not only a common oncogenic driver in lung adenocarcinoma but also a strong predisposing factor for the development of BM (3).

The advent of third-generation EGFR tyrosine kinase inhibitors (TKIs), such as osimertinib, aumolertinib, and furmonertinib, has revolutionized the management of EGFR-mutant NSCLC. These agents demonstrate superior systemic efficacy and, crucially, enhanced central nervous system (CNS) penetration compared to earlier-generation TKIs, leading to improved outcomes for patients with BM (4–7). Landmark trials like FLAURA and AENEAS have established these drugs as the standard first-line therapy, achieving impressive intracranial objective response rates and prolonging progression-free survival (8, 9). Despite these advances, a significant proportion of patients ultimately experience intracranial progression (10). Consequently, the optimal integration of local brain-directed therapy with potent systemic agents remains a critical and unresolved clinical question. Historically, whole-brain radiotherapy (WBRT) was the cornerstone of BM management but is associated with substantial neurocognitive sequelae (11). Modern techniques, such as stereotactic radiosurgery (SRS) and hippocampal-avoidance WBRT, aim to preserve cognitive function while providing effective local control (12, 13).

Prior studies evaluating the combination of first- or second-generation EGFR-TKIs with brain radiotherapy have yielded conflicting results regarding survival benefit, creating clinical equipoise (14–16). In the era of third-generation TKIs, the value of upfront radiotherapy is even more debated. While these drugs have high intracranial activity, some retrospective analyses suggest that deferring radiotherapy until progression does not compromise overall survival. Conversely, other data indicate that upfront SRS may delay intracranial failure and possibly improve survival outcomes (17–19). This controversy is compounded by a lack of studies specifically designed to compare distinct treatment strategies—namely, an intentional “early combined therapy” approach versus a “salvage radiotherapy” approach—in the first-line setting with modern agents and radiotherapy techniques.

Therefore, we conducted this multi-center retrospective study to directly compare the strategy of early combined brain radiotherapy with first-line third-generation EGFR-TKI against the strategy of initial TKI monotherapy followed by radiotherapy at intracranial progression. We hypothesized that the early combined strategy would be associated with superior intracranial control and overall survival, without a significant increase in treatment-related toxicity, and sought to identify patient subgroups most likely to benefit from this integrated approach.

## 2 Materials and methods

### 2.1 Patient selection and study design

This multi-center, retrospective cohort study was conducted at the Hebei Cangzhou Integrated Traditional Chinese and Western Medicine Hospital and the Tianjin Cancer Hospital. Consecutive patients with newly diagnosed EGFR-mutant NSCLC and synchronous brain metastases who initiated first-line therapy between January 2019 and July 2023 were screened for inclusion. The inclusion criteria were as follows: (1) histological or cytological confirmation of lung adenocarcinoma; (2) diagnosis of brain metastases supported by contrast-enhanced magnetic resonance imaging (MRI); (3) confirmation of a sensitizing EGFR mutation (exon 19 deletion or L858R point mutation) via next-generation sequencing or polymerase chain reaction-based testing of tumor tissue or plasma circulating tumor DNA; (4) treatment with a first-line third-generation EGFR-TKI (osimertinib, aumolertinib, or furmonertinib) with or without brain-directed radiotherapy; (5) age ≥ 18 years; and (6) availability of complete clinical and follow-up data. Key exclusion criteria included: (1) Eastern Cooperative Oncology Group (ECOG) performance status (PS) ≥ 3 at the initiation of first-line therapy; (2) diagnosis of leptomeningeal metastasis at baseline; (3) active pregnancy or lactation; (4) history of other active malignancies within the past 5 years; and (5) prior radiotherapy to the brain before the initiation of first-line TKI therapy. Based on the initial treatment strategy, eligible patients were classified into three groups for analysis: the Early Combined Therapy (ECT) group, the Salvage Radiotherapy (SRT) group, and the TKI Monotherapy (TKI-Mono) group. The specific definitions for group assignment are detailed in the subsequent section.

### 2.2 Treatment protocols

Patients were categorized into three groups based on the timing and intent of brain radiotherapy relative to first-line TKI initiation. Early Combined Therapy (ECT) Group: Patients who received brain radiotherapy within 90 days of starting first-line TKI, in the absence of intracranial progression. This represented a planned, upfront combined-modality strategy. Salvage Radiotherapy (SRT) Group: Patients who began first-line TKI alone and received brain radiotherapy only after radiographic confirmation of intracranial progression. This represented a deferred, as-needed salvage strategy. TKI Monotherapy (TKI-Mono) Group: Patients treated with first-line TKI alone, who did not receive any form of brain radiotherapy during the entire study follow-up period. Systemic Therapy: All patients received a standard dose of a third-generation EGFR-TKI: osimertinib (80 mg orally once daily), aumolertinib (110 mg orally once daily), or furmonertinib (80 mg orally once daily). Treatment continued until disease progression, unacceptable toxicity, or patient withdrawal. Radiotherapy Technique: All radiotherapy was planned using a dedicated treatment planning system (TPS) with CT simulation and co-registration of contrast-enhanced brain MRI for target delineation. For patients with 1–3 brain metastases (oligometastatic disease), stereotactic radiosurgery/radiotherapy (SRS/SRT) was employed. The planning target volume (PTV) was generated by adding a 3 mm margin to the gross tumor volume (GTV). The prescription isodose line was typically 70%-90%. Prescribed doses ranged from 30 to 45 Gy in 5 to 10 fractions (median 5 fractions), with the specific regimen chosen based on tumor size, location, and physician judgment, aiming to cover ≥95% of the PTV. For patients with >3 brain metastases, hippocampal-avoidance whole-brain radiotherapy (HA-WBRT) with a simultaneous integrated boost (SIB) to individual metastases was used. The whole brain (excluding hippocampi) was treated to 30 Gy in 10 fractions (3 Gy/fraction). Individual metastases received a simultaneous integrated boost; the boost dose prescribed to the GTV of metastases ranged from 10 to 20 Gy in 5–10 fractions (2 Gy/fraction). Delineation, Constraints, and Quality Assurance: Hippocampal contouring followed the RTOG 0933 protocol guidelines (20). A comprehensive set of dosimetric and clinical parameters was collected for all radiotherapy courses to address potential variability. This included: for SRS/SRT – prescription dose, number of fractions, biologically effective dose (BED10, assuming α/β=10), prescription isodose line, and conformity index; for HA-WBRT – whole brain dose, boost dose per metastasis, and mean/maximum dose to the hippocampi. Organs-at-risk (OAR) constraints were strictly applied: lens maximum dose (Dmax) <7 Gy; optic nerves/chiasm and brainstem Dmax <54 Gy; pituitary gland mean dose (Dmean) <45 Gy; and hippocampal Dmax maintained between 9–16 Gy as per protocol, with a goal of Dmean ≤10 Gy. The anatomic location of each brain metastasis (supratentorial vs. infratentorial) was also documented. All treatment plans underwent review and approval by a multidisciplinary tumor board including a specialized neuro-radiation oncologist. These detailed radiotherapy parameters are summarized for the ECT and SRT groups in **Supplementary Table 1**.

### 2.3 Outcome measures and response assessment

Efficacy Endpoints: The primary endpoint of this study was intracranial progression-free survival (iPFS), defined as the time from the initiation of first-line TKI therapy to intracranial disease progression as per Response Evaluation Criteria in Solid Tumors (RECIST) version 1.1 (21), or death from any cause, whichever occurred first. Key secondary endpoints included: overall survival (OS), measured from treatment initiation to death from any cause; progression-free survival (PFS), defined as the time to systemic or intracranial progression or any-cause death; intracranial objective response rate (iORR), calculated as the proportion of patients achieving a complete response (CR) or partial response (PR) as the best intracranial response; and intracranial disease control rate (iDCR), defined as the proportion of patients with a best response of CR, PR, or stable disease (SD) lasting for at least 6 weeks. Radiologic and Response Assessment: Tumor response was evaluated using contrast-enhanced computed tomography (CT) of the chest and abdomen and contrast-enhanced MRI of the brain. Baseline imaging was performed within 4 weeks before treatment initiation. Follow-up assessments were conducted every 8–12 weeks thereafter, or earlier if clinically indicated. Intracranial and extracranial responses were evaluated separately according to RECIST 1.1 criteria by two independent radiologists, with any discrepancies resolved by consensus. Safety and Toxicity Assessment: Treatment-related adverse events (AEs) were graded throughout the treatment and follow-up period according to the National Cancer Institute’s Common Terminology Criteria for Adverse Events (CTCAE) version 5.0 (22). Specific attention was paid to neurologic toxicities. Symptomatic radiation necrosis was diagnosed based on clinical symptoms correlated with characteristic imaging findings and requiring medical intervention. Neurocognitive function was assessed using the Mini-Mental State Examination (MMSE) at baseline (23), and at 1, 3, and 6 months after the completion of radiotherapy for patients in the ECT and SRT groups. A decline of ≥3 points from baseline was considered clinically significant cognitive deterioration. Late radiographic changes were evaluated using the Fazekas scale for white matter lesions on T2/FLAIR MRI sequences.

### 2.4 Follow-up

Patients were followed from the initiation of first-line therapy until death, loss to follow-up, or the study cutoff date of January 31, 2025. Follow-up was conducted through regular clinical visits, inpatient records, and telephone interviews. Clinical, imaging, and laboratory data were collected at each visit. Survival status was ascertained from hospital records or, when necessary, through direct contact with patients or their families. The median follow-up duration was calculated using the reverse Kaplan-Meier estimator.

### 2.5 Multi-center considerations and treatment homogeneity

This study involved two tertiary care institutions. While treatment decisions (ECT vs. SRT) were made historically by local multidisciplinary teams, we implemented a retrospective harmonization process. All cases were re-evaluated against the pre-defined study criteria to ensure consistent group assignment. Both centers utilized modern radiotherapy techniques (SRS/SRT and HA-WBRT with hippocampal avoidance) and followed national guidelines for systemic therapy. To assess the potential for center-specific bias, we compared the distribution of key baseline characteristics and the selection of radiotherapy technique between centers. No major systematic differences were identified that would compromise the validity of pooling data for the primary comparative analysis.

### 2.6 Statistical analysis

Statistical analyses were performed using SPSS (version 26.0) and R (version 4.3.1). Categorical variables were compared with the Chi-square or Fisher’s exact test, and continuous variables with the Mann-Whitney U or Kruskal-Wallis test. Survival curves were generated using the Kaplan-Meier method and compared with the log-rank test. Hazard ratios (HR) and 95% confidence intervals (CI) were derived from Cox proportional hazards models, both univariable and multivariable. A two-sided P value < 0.05 was considered statistically significant.

## 3 Results

### 3.1 Patient characteristics and group comparability

A total of 193 patients with treatment-naïve EGFR-mutant NSCLC and brain metastases were included in this retrospective analysis. Based on the initial treatment strategy, patients were stratified into three cohorts: the ECT group (n=83), the SRT group (n=83), and the TKI-Mono (n=27). The baseline demographic and clinical characteristics of the three groups are summarized in **Table 1**. No statistically significant differences were observed among the groups regarding sex, age, smoking history, KPS, comorbidities, EGFR mutation subtype, presence of extracranial metastases, number and size of brain metastases, presence of baseline intracranial symptoms, or the choice of first-line third-generation EGFR-TKI (all P > 0.05). The distribution of radiation technique between the ECT and SRT groups showed no significant difference (P = 0.080). The overall comparability of baseline features supports the validity of subsequent comparative efficacy analyses.

**Table 1:** Baseline demographic and clinical characteristics of the study population.

Characteristic	ECT group (n=83)	SRT group (n=83)	TKI-mono group (n=27)	χ² Value	P Value
Sex				0.517	0.772
Male	42 (50.6)	38 (45.8)	12 (44.4)		
Female	41 (49.4)	45 (54.2)	15 (55.6)		
Age, years				1.796	0.407
< 65	52 (62.7)	48 (57.8)	13 (48.1)		
≥ 65	31 (37.3)	35 (42.2)	14 (51.9)		
Smoking History				0.625	0.731
Yes	28 (33.7)	32 (38.6)	11 (40.7)		
No	55 (66.3)	51 (61.4)	16 (59.3)		
KPS				1.978	0.372
≥ 80	75 (90.4)	70 (84.3)	22 (81.5)		
< 80	8 (9.6)	13 (15.7)	5 (18.5)		
Comorbidities				1.610	0.447
Yes	35 (42.2)	40 (48.2)	15 (55.6)		
No	48 (57.8)	43 (51.8)	12 (44.4)		
EGFR Mutation				0.027	0.987
Exon 19 deletion	43 (51.8)	44 (53.0)	14 (51.9)		
L858R	40 (48.2)	39 (47.0)	13 (48.1)		
Extracranial Metastasis at Diagnosis				1.760	0.415
Yes	47 (56.6)	52 (62.7)	19 (70.4)		
No	36 (43.4)	31 (37.3)	8 (29.6)		
Number of Brain Metastases				1.734	0.420
1-3	58 (69.9)	50 (60.2)	17 (63.0)		
>3	25 (30.1)	33 (39.8)	10 (37.0)		
Largest Brain Metastasis Diameter				1.620	0.445
≤ 2 cm	45 (54.2)	40 (48.2)	11 (40.7)		
> 2 cm	38 (45.8)	43 (51.8)	16 (59.3)		
Baseline Intracranial Symptoms				3.194	0.203
Yes	30 (36.1)	35 (42.2)	15 (55.6)		
No	53 (63.9)	48 (57.8)	12 (44.4)		
First-line TKI Agent				0.207	0.995
Osimertinib	53 (63.9)	55 (66.3)	18 (66.7)		
Aumolertinib	18 (21.7)	17 (20.5)	5 (18.5)		
Furmonertinib	12 (14.4)	11 (13.2)	4 (14.8)		
Radiation Technique^				3.060	0.080
Stereotactic Radiosurgery/Radiotherapy	56 (67.5)	45 (54.2)	N/A		
Whole-Brain Radiotherapy ± SIB	27 (32.5)	38 (45.8)	N/A		

Data are presented as n (%). ECT, Early Combined Therapy; SRT, Salvage Radiotherapy; TKI-Mono, TKI Monotherapy; KPS, Karnofsky Performance Status; SIB, Simultaneous Integrated Boost; ^Radiation technique was compared only between the ECT and SRT groups; N/A, Not applicable.

### 3.2 Survival outcomes

**Figure 1:**
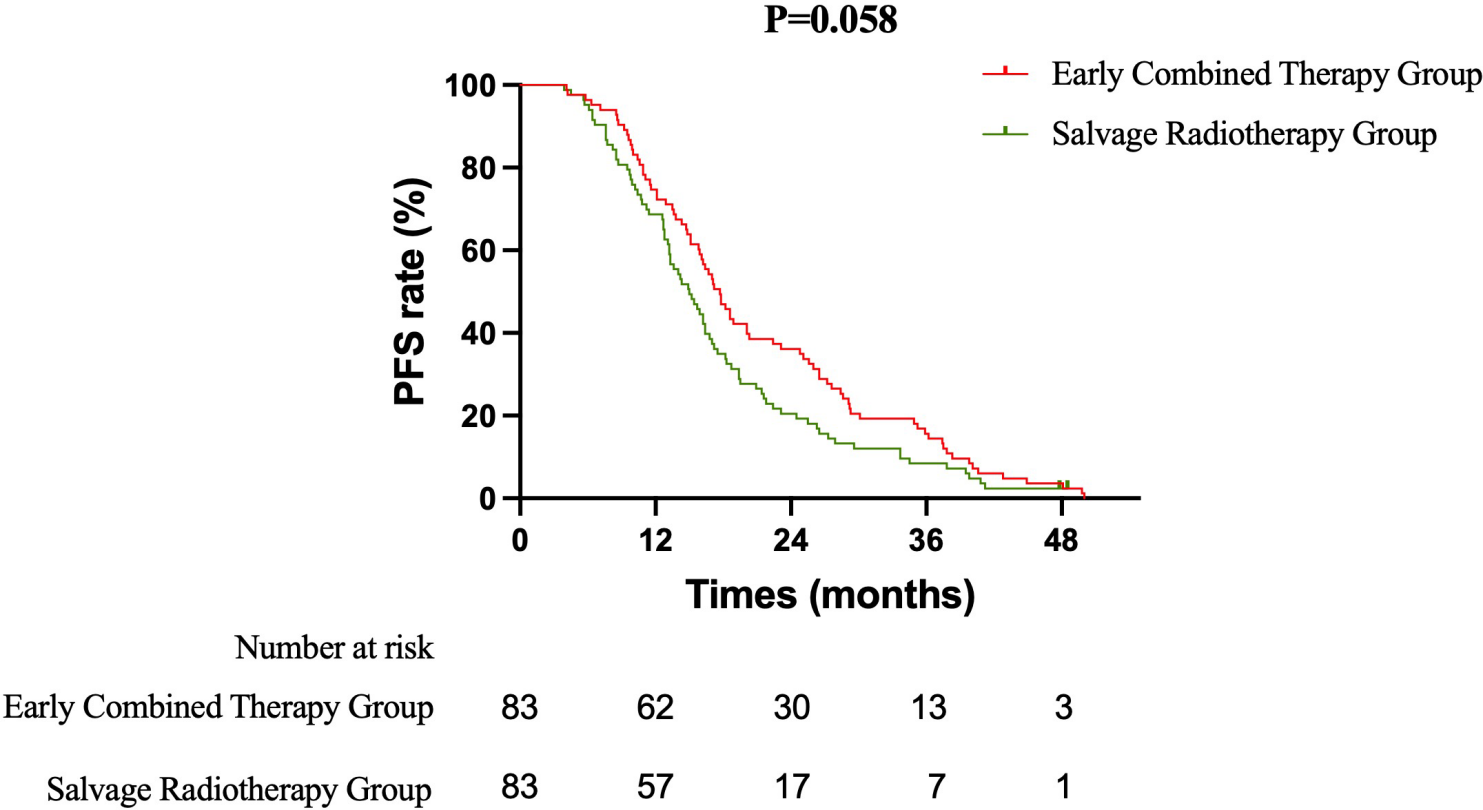
Kaplan-Meier curves for overall survival (OS) comparing the Early Combined Therapy (ECT) and Salvage Radiotherapy (SRT) groups.

**Figure 2:**
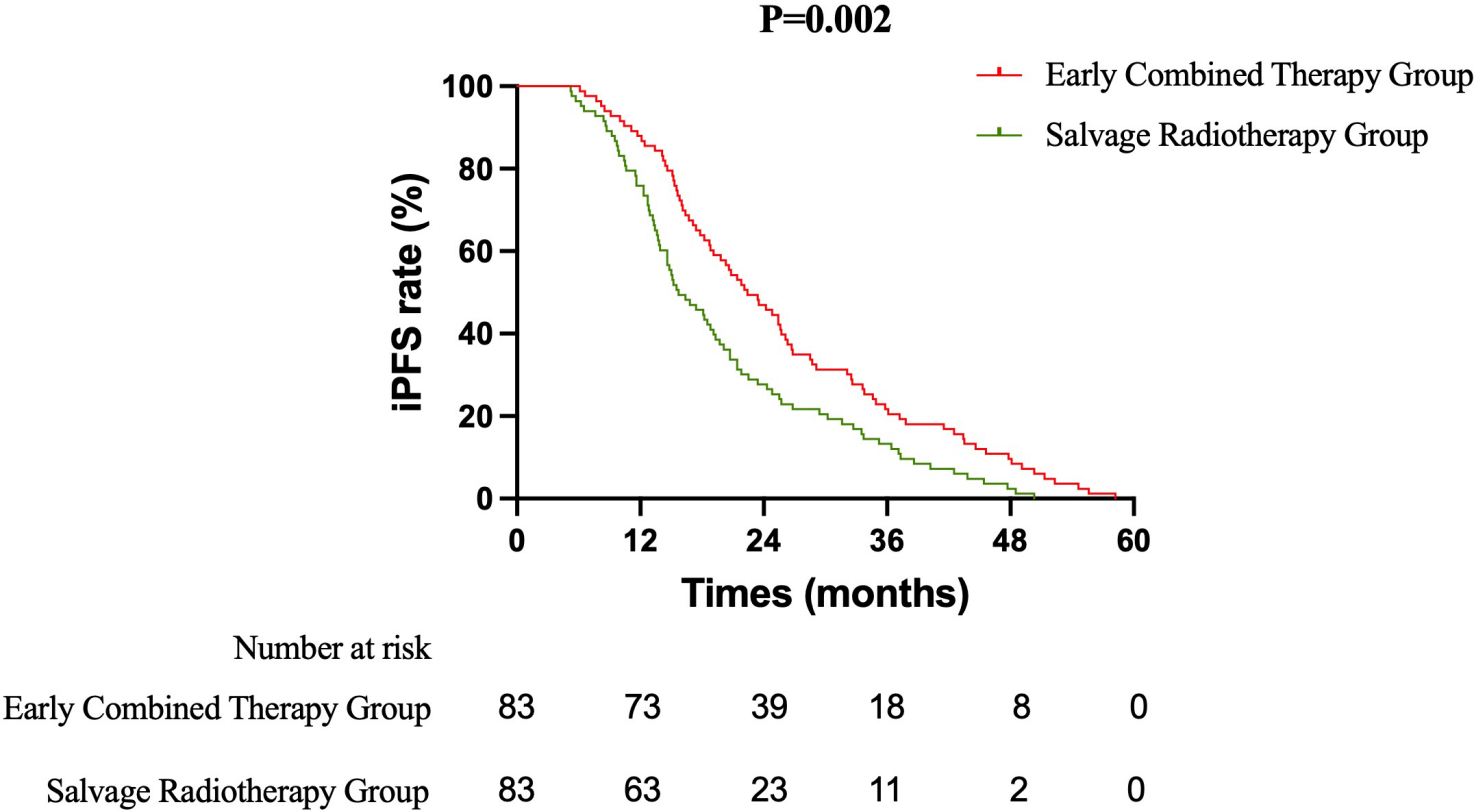
Kaplan-Meier curves for intracranial progression-free survival (iPFS) comparing the Early Combined Therapy (ECT) and Salvage Radiotherapy (SRT) groups.

**Figure 3:**
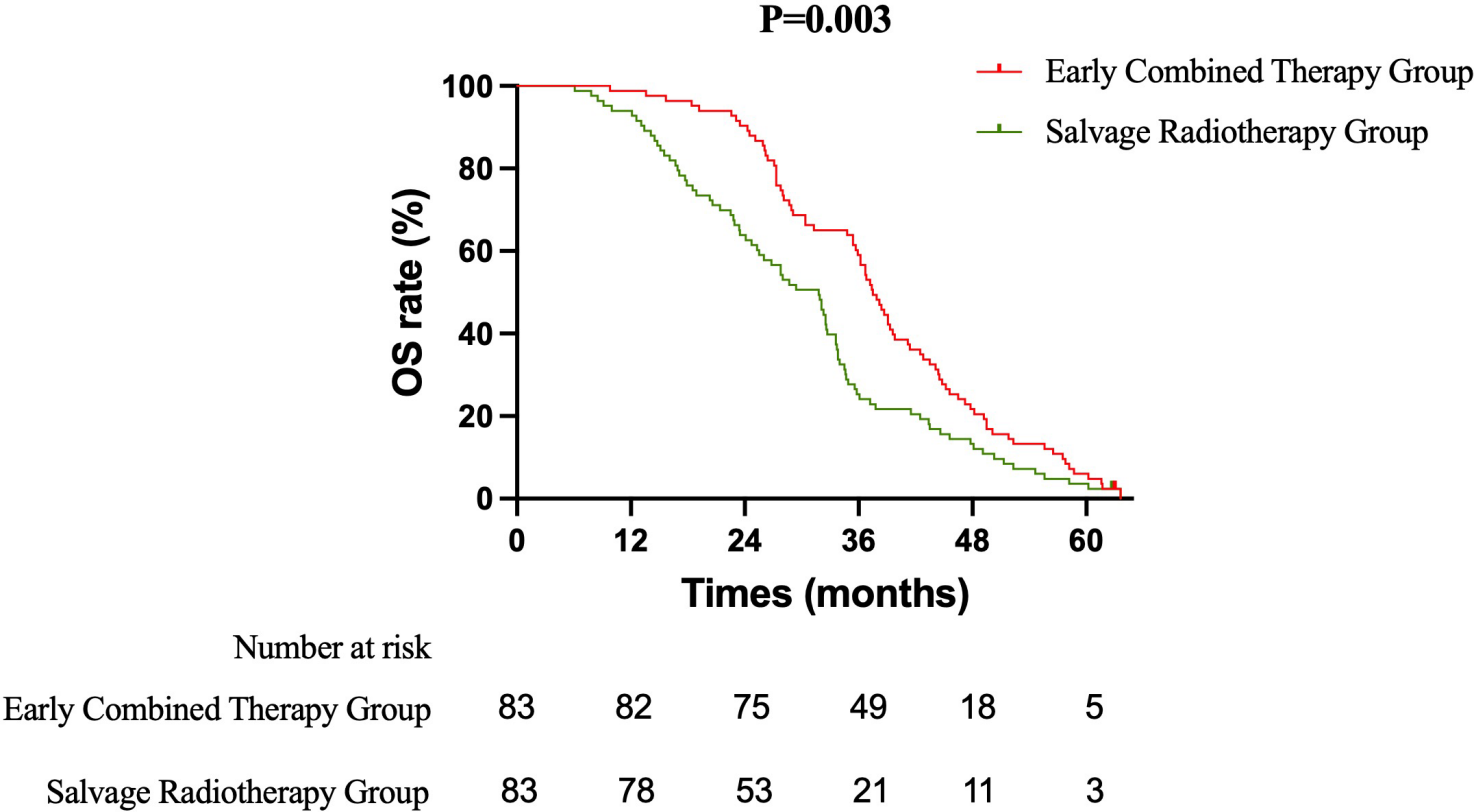
Kaplan-Meier curves for progression-free survival (PFS) comparing the Early Combined Therapy (ECT) and Salvage Radiotherapy (SRT) groups.

**Figure 4:**
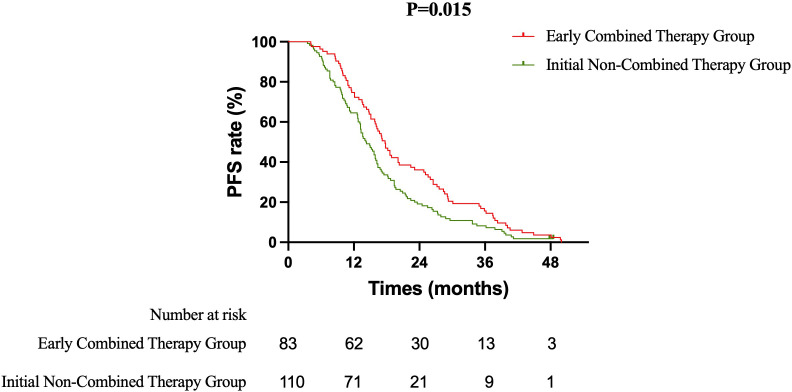
Kaplan-Meier curves for overall survival (OS) comparing the Early Combined Therapy (ECT) group with the combined Initial Non-Combined Therapy cohort (SRT + TKI-Mono).

**Figure 5:**
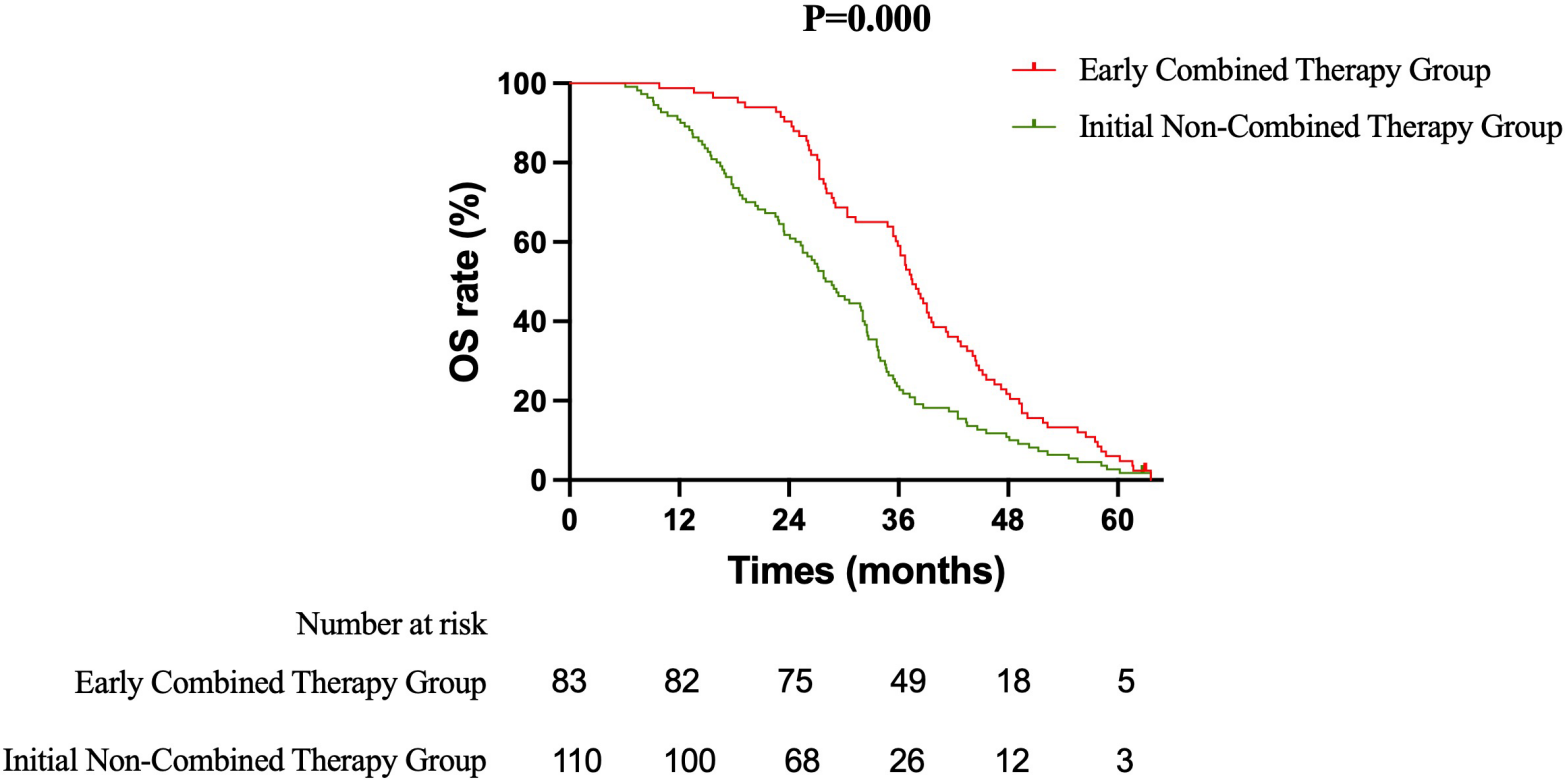
Kaplan-Meier curves for intracranial progression-free survival (iPFS) comparing the Early Combined Therapy (ECT) group with the combined Initial Non-Combined Therapy cohort (SRT + TKI-Mono).

**Figure 6:**
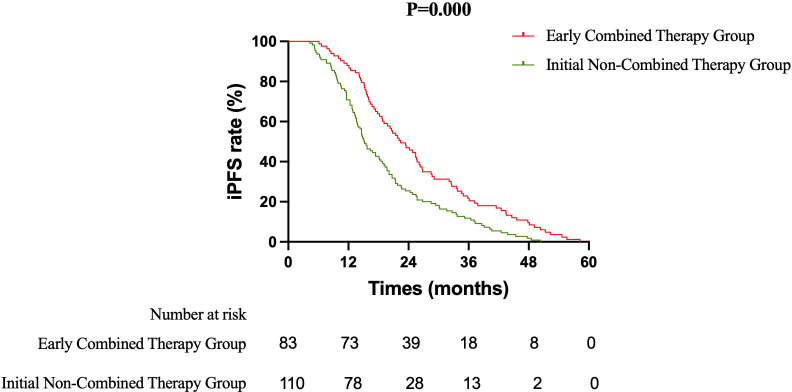
Kaplan-Meier curves for progression-free survival (PFS) comparing the Early Combined Therapy (ECT) group with the combined Initial Non-Combined Therapy cohort (SRT + TKI-Mono).

Primary Efficacy Analysis ECT versus SRT: The primary survival comparison between the ECT and SRT strategies demonstrated superior efficacy for the ECT approach across key endpoints. The median OS was significantly longer in the ECT group than in the SRT group (37.5 months vs. 31.8 months; HR 0.637, 95% CI 0.465–0.871; log-rank P = 0.003) (**Figure 1**). The benefit in intracranial disease control was particularly pronounced. The median iPFS was 22.4 months in the ECT group compared to 15.7 months in the SRT group (HR 0.628, 95% CI 0.459–0.858; P = 0.002) (**Figure 2**). A consistent, though less pronounced, trend favoring the ECT strategy was observed for systemic PFS, with median PFS of 17.7 months versus 15.0 months (HR 0.748, 95% CI 0.549–1.020; P = 0.058) (**Figure 3**). Sensitivity Analysis: ECT versus Initial Non-Combined Therapy: To assess the robustness of the primary findings, a sensitivity analysis was performed by combining the SRT group with the TKI-Mono group (n=27; median OS 26.5 months, median iPFS 13.6 months, median PFS 13.2 months) into an “Initial Non-Combined Therapy” cohort (total n=110). The survival advantage of the ECT strategy remained significant and was numerically enhanced in this comparison. The ECT group maintained a superior median OS compared to the combined cohort (37.5 months vs. 28.7 months; HR 0.589, 95% CI 0.442–0.784; P = 0.000) (**Figure 4**). Similarly, the iPFS benefit persisted (median 22.4 months vs. 15.2 months; HR 0.590, 95% CI 0.444–0.784; P = 0.000) (**Figure 5**). The PFS difference also reached statistical significance in this analysis (median 17.7 months vs. 14.3 months; HR 0.706, 95% CI 0.531–0.937; P = 0.015) (**Figure 6**).

### 3.3 Intracranial tumor response

The iORR and iDCR following treatment are detailed in **Table 2**. The iORR was significantly higher in the ECT group compared to the SRT group (81.9% vs. 66.3%, P = 0.021). In the TKI-Mono group, the iORR was 59.3%. The overall difference in iORR across all three groups was also statistically significant (P = 0.022). The iDCR was high in all groups, reaching 100% in the ECT group, 97.6% in the SRT group, and 96.3% in the TKI-Mono group. No significant difference in iDCR was observed between the ECT and SRT groups (P = 0.497) or across the three groups collectively (P = 0.363).

**Table 2:** Best intracranial response according to RECIST 1.1 criteria.

Response	ECT group (n=83)	SRT group (n=83)	TKI-mono group (n=27)	χ² value	P value
CR	15 (18.1)	9 (10.8)	3 (11.1)		
PR	53 (63.9)	46 (55.4)	13 (48.1)		
SD	15 (18.1)	26 (31.3)	10 (37.0)		
PD	0 (0.0)	2 (2.4)	1 (3.7)		
iORR (CR+PR)	68 (81.9)	55 (66.3)	16 (59.3)	7.589	0.022
iDCR (CR+PR+SD)	83 (100)	81 (97.6)	26 (96.3)	2.918	0.363

Data are presented as n (%). iORR, intracranial objective response rate; iDCR, intracranial disease control rate; RECIST, Response Evaluation Criteria in Solid Tumors; ECT, Early Combined Therapy; SRT, Salvage Radiotherapy; TKI-Mono, TKI Monotherapy.

### 3.4 Multivariate analyses of prognostic factors

To identify independent prognostic factors, multivariate Cox proportional hazards analyses were performed for OS and iPFS, adjusting for key baseline and treatment-related variables, including radiotherapy technique (SRS/SRT vs. HA-WBRT). The results are presented in **Tables 3**, **4**. In the multivariate model for OS (**Table 3**), the ECT strategy remained an independent factor associated with significantly improved survival after adjustment for other variables (adjusted HR 0.602, P = 0.009). The only other independent prognostic factor was a higher number of brain metastases (>3), which was associated with worse OS (adjusted HR 1.852, P<0.001). The analysis for iPFS yielded consistent and stronger results (**Table 4**). The ECT strategy was independently associated with a markedly reduced risk of intracranial progression (adjusted HR 0.523, P<0.001). Additionally, the use of stereotactic radiotherapy (vs. WBRT) was associated with superior intracranial control (adjusted HR 0.621, P = 0.009). As expected, a higher number of brain metastases was the strongest independent adverse prognostic factor for iPFS (adjusted HR 2.215, P<0.001). The type of EGFR-TKI used, age, EGFR mutation subtype, and presence of extracranial metastases were not independent prognostic factors for either endpoint in the adjusted models.

**Table 3:** Multivariate Cox regression analysis for OS.

Variable	Level	Adjusted HR	95% CI	P value
Treatment Strategy	ECT vs. SRT	0.602	0.412 – 0.880	0.009
Radiation Technique	SRS/SRT vs. WBRT ± SIB	0.898	0.490 – 1.995	0.747
First-line TKI Agent	Aumolertinib vs. Osimertinib	1.050	0.672 – 1.642	0.826
	Furmonertinib vs. Osimertinib	0.943	0.581 – 1.531	0.810
Age	≥65 vs. <65 years	1.321	0.938 – 1.861	0.112
EGFR Mutation	L858R vs. Exon 19 deletion	1.277	0.909 – 1.794	0.157
Number of Brain Metastases	>3 vs. 1-3	1.852	1.301 – 2.637	<0.001
Extracranial Metastasis	Present vs. Absent	1.423	0.998 – 2.029	0.051

Analysis based on the ECT and SRT groups (n=166). ECT, Early Combined Therapy; SRT, Salvage Radiotherapy; SRS/SRT, Stereotactic Radiosurgery/Radiotherapy; WBRT ± SIB, Whole-Brain Radiotherapy with or without Simultaneous Integrated Boost.

**Table 4:** Multivariate Cox regression analysis for intracranial iPFS.

Variable	Level	Adjusted HR	95% CI	P value
Treatment Strategy	ECT vs. SRT	0.523	0.358 – 0.764	<0.001
Radiation Technique	SRS/SRT vs. WBRT ± SIB	0.621	0.435 – 0.886	0.009
First-line TKI Agent	Aumolertinib vs. Osimertinib	1.102	0.705 – 1.722	0.670
	Furmonertinib vs. Osimertinib	0.985	0.607 – 1.600	0.952
Age	≥65 vs. <65 years	1.189	0.843 – 1.677	0.321
EGFR Mutation	L858R vs. Exon 19 deletion	1.358	0.967 – 1.908	0.077
Number of Brain Metastases	>3 vs. 1-3	2.215	1.556 – 3.154	<0.001
Extracranial Metastasis	Present vs. Absent	1.207	0.846 – 1.723	0.301

Analysis based on the ECT and SRT groups (n=166). ECT, Early Combined Therapy; SRT, Salvage Radiotherapy; SRS/SRT, Stereotactic Radiosurgery/Radiotherapy; WBRT ± SIB, Whole-Brain Radiotherapy with or without Simultaneous Integrated Boost.

### 3.5 Subgroup analyses

**Figure 7:**
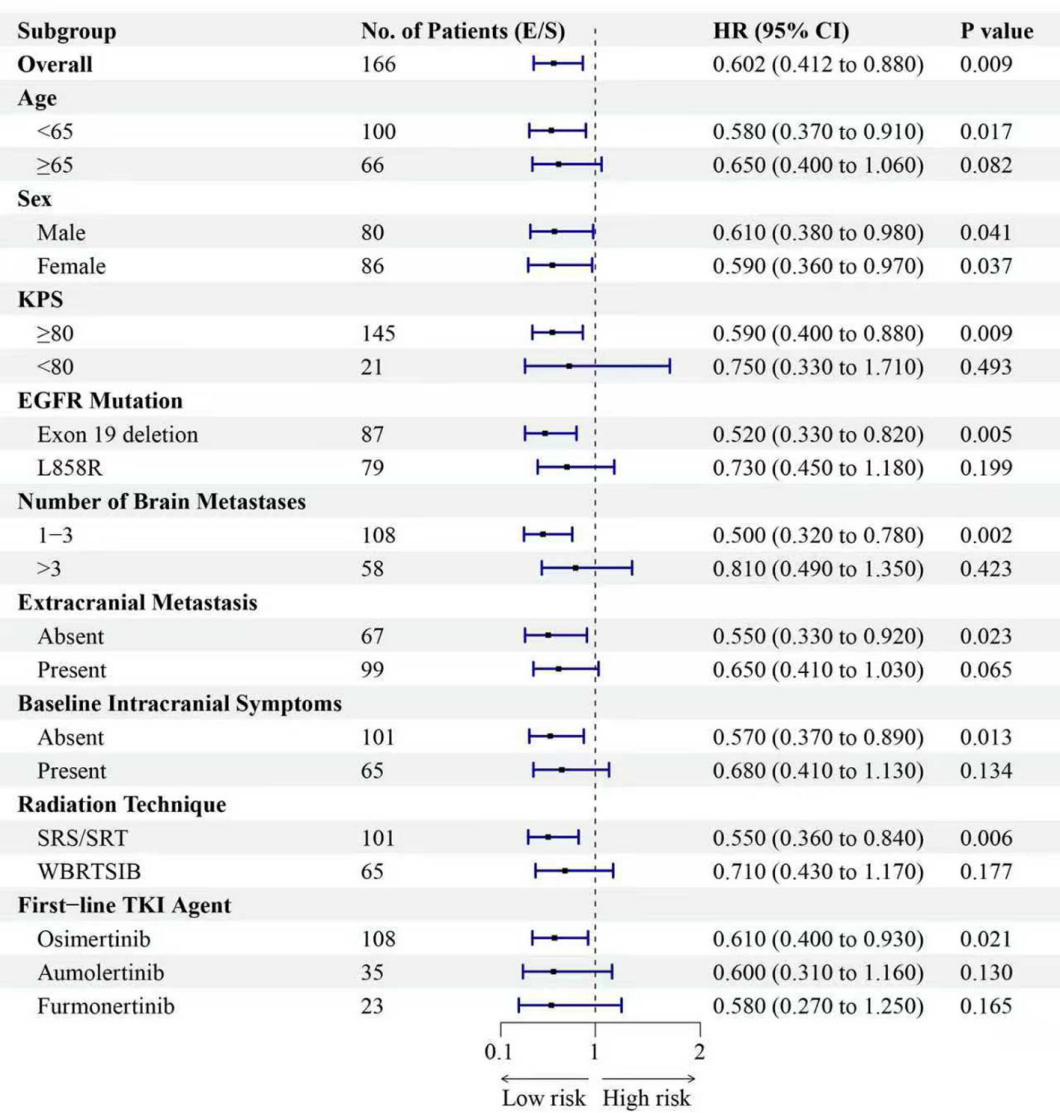
Forest plot of subgroup analyses for overall survival (OS) favoring the Early Combined Therapy (ECT) strategy. Presented are hazard ratios and 95% confidence intervals across various baseline characteristics.

**Figure 8:**
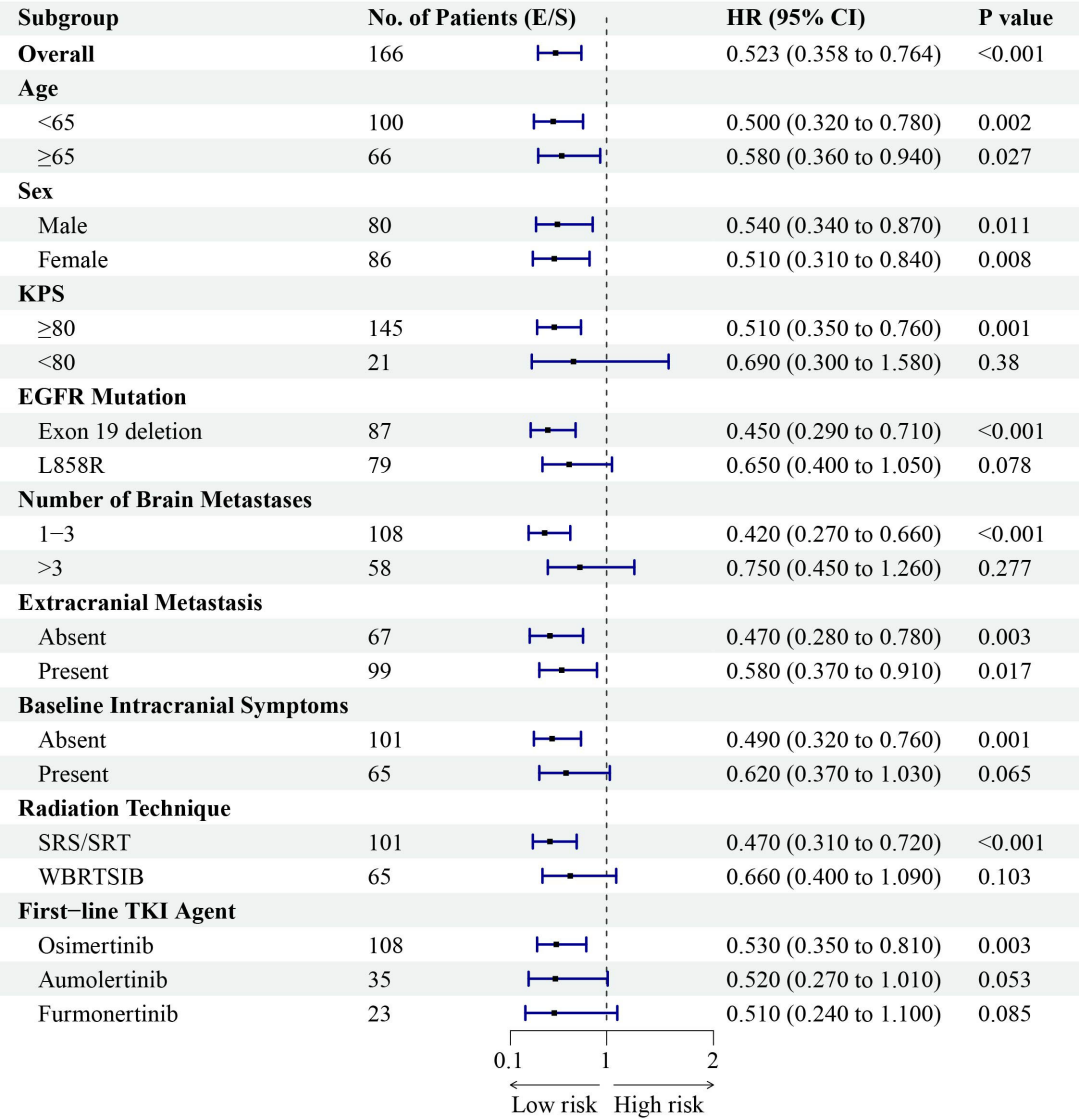
Forest plot of subgroup analyses for intracranial progression-free survival (iPFS) favoring the Early Combined Therapy (ECT) strategy. Presented are hazard ratios and 95% confidence intervals across various baseline characteristics.

Subgroup analyses consistently favored the ECT strategy across most patient categories for both OS and iPFS (**Figures 7**, **8**). The survival benefit was particularly pronounced in key clinical subgroups. Patients with EGFR exon 19 deletion derived greater benefit in both OS (HR 0.52, P = 0.005) and iPFS (HR 0.45, P<0.001) compared to those with the L858R mutation. Similarly, the advantage of the ECT strategy was most significant among patients with 1–3 brain metastases for OS (HR 0.50, P = 0.002) and iPFS (HR 0.42, P<0.001). A consistent treatment effect was observed across other subgroups, including those defined by age, sex, KPS, and the presence of extracranial metastases. To visually complement the subgroup analyses and further assess the impact of intracranial tumor burden, we performed additional stratified analyses based on the number of brain metastases (1–3 vs. >3) and the largest brain metastasis diameter (<1 cm vs. ≥1 cm). Kaplan-Meier curves comparing the three treatment strategies (ECT, SRT, and TKI-Mono) within each stratum are presented in **Supplementary Figures 1**-**8**. Analysis by Number of Brain Metastases: Among patients with 1–3 brain metastases, the ECT group demonstrated significantly longer iPFS (median 25.1 months) and OS (median 38.9 months) compared to both the SRT (iPFS: 18.7 months; OS: 32.6 months) and TKI-Mono groups (iPFS: 14.7 months; OS: 27.2 months; P = 0.001 for both iPFS and OS) (**Supplementary Figures 1**, **2**). In patients with >3 brain metastases, the ECT strategy was also associated with significantly longer iPFS (median 15.8 vs. 13.8 vs. 10.1 months, P = 0.027) and a non-significant trend toward longer OS (median 28.1 vs. 23.5 vs. 19.4 months, P = 0.071) compared to SRT and TKI-Mono, respectively (**Supplementary Figures 3**, **4**). Analysis by Largest Brain Metastasis Diameter: Similarly, in the subgroup with the largest brain metastasis diameter <1 cm, the ECT group had significantly improved iPFS (median 22.3 months) and OS (median 37.5 months) compared to the SRT (iPFS: 14.6 months; OS: 27.8 months) and TKI-Mono groups (iPFS: 11.6 months; OS: 21.3 months; P = 0.014 and P = 0.042, respectively) (**Supplementary Figures 5**, **6**). For patients with lesions ≥1 cm, ECT was associated with significantly longer iPFS (median 23.4 vs. 16.8 vs. 13.6 months, P = 0.006) and a trend toward longer OS (median 37.9 vs. 32.1 vs. 26.5 months, P = 0.090) (**Supplementary Figures 7**, **8**).

### 3.6 Radiotherapy parameters and outcomes by technique

Detailed radiotherapy parameters for the ECT and SRT groups are summarized in **Supplementary Table 1**, demonstrating overall comparability in prescription doses, fractionation schemes, and adherence to organs-at-risk constraints between the two strategy groups. Exploratory Analysis of Radiotherapy Technique: To address the potential impact of radiotherapy modality, we first compared outcomes between patients who received SRS/SRT (n=101) and those who received HA-WBRT (n=65), irrespective of treatment timing. Patients treated with SRS/SRT had significantly longer iPFS than those receiving HA-WBRT (median 21.4 vs. 16.4 months; HR 0.716, 95% CI 0.516–0.993; P = 0.033) (**Supplementary Figure 9**). However, there was no significant difference in OS between the technique groups (median 34.5 vs. 32.1 months; HR 0.885, 95% CI 0.645–1.215; P = 0.435) (**Supplementary Figure 10**) or in systemic PFS (median 17.0 vs. 14.0 months; HR 0.834, 95% CI 0.606–1.148; P = 0.246) (**Supplementary Figure 11**). Effect of Treatment Strategy Within Radiotherapy Subgroups: We next examined whether the benefit of the ECT strategy was consistent across the different radiotherapy modalities through pre-specified subgroup analysis (**Figures 7**, **8**). The survival advantage of ECT over SRT was significant within the subgroup of patients treated with SRS/SRT for both OS (HR 0.55, 95% CI 0.360–0.840; P = 0.006) and iPFS (HR 0.47, 95% CI 0.310–0.720; P<0.001). In the HA-WBRT subgroup, a trend favoring ECT was observed, but it did not reach statistical significance for OS (HR 0.71, 95% CI 0.430–1.170; P = 0.177) or iPFS (HR 0.66, 95% CI 0.400–1.090; P = 0.103).

### 3.7 Treatment-related adverse events

The safety profile of the ECT strategy was comparable to that of the SRT strategy. As detailed in **Table 5**, the incidence of grade 3 or higher AEs was similar between the ECT and SRT groups (33.7% vs. 30.1%, P = 0.617). The spectrum of AEs was consistent with the known profiles of third-generation EGFR-TKIs and cranial radiotherapy. No significant between-group differences were observed in the rates of specific TKI-related toxicities (e.g., rash, diarrhea), acute radiotherapy-related events (e.g., headache, nausea), or hematologic toxicities (all P>0.05). Of critical importance, the incidence of key neurologic toxicities was low and did not differ between the two strategies. Symptomatic radiation necrosis occurred in 2.4% of ECT and 1.2% of SRT patients (P = 1.000). Similarly, the rates of symptomatic cerebral edema requiring steroid intervention (3.6% vs. 4.8%, P = 1.000) and objective cognitive decline (4.8% vs. 6.0%, P = 1.000) were comparable.

**Table 5:** Incidence of grade ≥3 treatment-related adverse events.

Adverse event (CTCAE v5.0)	ECT group (n=83)	SRT group (n=83)	χ² value	P value
Any Grade ≥3 AE	28 (33.7)	25 (30.1)	0.249	0.617
TKI-Related AEs				
Rash	6 (7.2)	5 (6.0)	0.097	0.755
Diarrhea	4 (4.8)	3 (3.6)	–	1.000
Stomatitis	3 (3.6)	2 (2.4)	–	1.000
Paronychia	2 (2.4)	1 (1.2)	–	1.000
Hepatic Dysfunction (ALT/AST increase)	2 (2.4)	3 (3.6)	–	1.000
Radiotherapy-Related AEs				
Headache	4 (4.8)	5 (6.0)	–	1.000
Nausea/Vomiting	3 (3.6)	6 (7.2)	–	0.496
Fatigue	7 (8.4)	5 (6.0)	0.359	0.549
Hematologic Toxicity				
Leukopenia	9 (10.8)	6 (7.2)	0.660	0.417
Neutropenia	8 (9.6)	5 (6.0)	0.751	0.386
Thrombocytopenia	6 (7.2)	5 (6.0)	0.097	0.755
Key Neurologic Toxicities				
Symptomatic Radiation Necrosis	2 (2.4)	1 (1.2)	–	1.000
Symptomatic Cerebral Edema (requiring steroids)	3 (3.6)	4 (4.8)	–	1.000
Cognitive Decline (MMSE decrease ≥3 points)	4 (4.8)	5 (6.0)	–	1.000

Data are presented as n (%). CTCAE, Common Terminology Criteria for Adverse Events; ALT, Alanine Aminotransferase; AST, Aspartate Aminotransferase; MMSE, Mini-Mental State Examination; ECT, Early Combined Therapy; SRT, Salvage Radiotherapy.- P value derived from Fisher’s exact test.

## 4 Discussion

This multi-center retrospective study directly compared two contemporary management strategies for patients with EGFR-mutant NSCLC presenting with synchronous BMs in the first-line setting of third-generation EGFR-TKIs. Our principal findings demonstrate that an ECT strategy, integrating upfront brain-directed radiotherapy within 90 days of initiating a third-generation EGFR-TKI, was associated with significantly superior intracranial control and overall survival compared to a strategy of initial TKI monotherapy followed by SRT at progression. Notably, this survival benefit was achieved without a significant increase in severe toxicities or neurocognitive sequelae. These results contribute critical real-world evidence to the ongoing debate regarding the optimal integration of highly effective systemic therapy and modern radiotherapy for this patient population.

Our finding that upfront cranial radiotherapy prolongs both iPFS and OS aligns with the growing body of literature suggesting a synergistic or additive benefit from early local therapy, even in the era of CNS-penetrant TKIs. The study by Yu et al. (18) similarly reported that upfront cranial RT (primarily SRS for oligometastatic disease) significantly improved iPFS and OS in osimertinib-treated patients, particularly in those with oligometastatic BMs. This consistency strengthens the hypothesis that local consolidation of macroscopic disease may delay or prevent intracranial failure, a common site of initial progression. Furthermore, Zhao et al. (19) found that upfront SRS/surgery provided an additional OS benefit in patients receiving either first- or third-generation EGFR-TKIs, reinforcing the concept that local control remains a critical determinant of long-term outcomes. Our multivariate analysis corroborates this, identifying the ECT strategy as an independent favorable prognostic factor for OS and iPFS, alongside the use of stereotactic (vs. whole-brain) radiotherapy.

However, our results contrast with several studies that reported no OS benefit with upfront radiotherapy. The retrospective analysis by Wang et al. (24), while confirming superior iPFS with upfront RT (using earlier-generation TKIs), found no difference in OS, suggesting salvage RT for oligo-progression could effectively “catch up.” Similarly, Xie et al. (25) observed no difference in survival outcomes between patients receiving osimertinib with or without concurrent radiation for progressing BMs. The divergence from our findings may be attributed to several key methodological differences. First, our study specifically focused on first-line third-generation TKI use in treatment-naïve patients with synchronous BMs, defining a homogenous, contemporary cohort. Second, we employed a strict, clinically relevant definition for the “early combined” strategy (radiotherapy within 90 days, prior to progression), differentiating it from salvage intent. Third, the widespread adoption of hippocampal-avoidance techniques and SRS in our cohort likely mitigated the neurocognitive toxicity historically associated with WBRT, preserving the therapeutic ratio and potentially allowing the survival benefit of improved intracranial control to be fully realized. The low and comparable rates of cognitive decline and symptomatic necrosis between our ECT and SRT groups support this notion.

The subgroup analyses offer valuable insights for patient selection. The pronounced benefit of the ECT strategy in patients with 1–3 BMs and those harboring exon 19 deletions is particularly noteworthy. For oligometastatic disease, the rationale for aggressive local consolidation is strong, as SRS can deliver ablative doses with high precision and minimal toxicity. This aligns with the conclusions of Yu et al. (18). The differential benefit based on EGFR mutation subtype (exon 19 del vs. L858R) is an intriguing finding that warrants further investigation. While Zhai et al. (26) reported a conflicting interaction (OS benefit with RT in L858R but not in exon 19 del), heterogeneity in study populations, sample sizes, and radiotherapy techniques may account for these discrepancies. Our data suggest that the more aggressive biology potentially associated with exon 19 deletions might be particularly amenable to upfront multimodal attack. This hypothesis-generating observation underscores the need for biomarker-driven strategies (27).

The detailed analysis of radiotherapy parameters further refines the interpretation of our findings. Our exploratory comparison revealed that the stereotactic technique (SRS/SRT) itself was associated with superior intracranial control compared to hippocampal-avoidance WBRT, a result consistent with the established dose-response relationship for ablative radiotherapy (13). However, this technical advantage did not translate into an overall survival difference, underscoring the dominant impact of systemic disease biology and the effectiveness of subsequent salvage therapies. More importantly, the pre-specified subgroup analysis demonstrated that the survival benefit of the early combined strategy was most evident and statistically significant among patients who received SRS/SRT. This observation suggests that the timing of radiotherapy integration (early versus salvage) and the technique employed (focal versus whole-brain) are interdependent factors. The most favorable outcomes may be achieved when an early, combined modality approach is applied with precise, focal techniques like SRS/SRT, which maximize local control while minimizing neurocognitive risk. The trend favoring early combination in the HA-WBRT subgroup, albeit not statistically significant, indicates that the strategy may still hold value in patients with more numerous metastases, though this requires validation in larger cohorts.

The excellent safety profile observed is a cornerstone of our argument for the ECT strategy. The incidence of high-grade AEs and specific neurotoxicities was low and comparable between groups. This challenges the historical concern that combining radiotherapy with TKI inevitably amplifies toxicity. Our protocol, which mandated modern techniques like HA-WBRT with SIB for multiple metastases and strict organ-at-risk constraints, demonstrates that such combinations are feasible and well-tolerated in real-world practice. This finding is consistent with the toxicity assessment by Yang et al. (28), who also reported well-tolerated profiles for both upfront and deferred RT sequences.

Several limitations of our study must be acknowledged. First, as a retrospective study conducted across two centers, differences in institutional practice patterns and physician preference could have influenced the choice between treatment strategies (ECT vs. SRT). Although we implemented a retrospective harmonization process for study definitions and observed a consistent direction of treatment effect across centers, unmeasured confounding related to center-specific factors may persist. Second, its retrospective nature introduces inherent selection bias, despite balanced baseline characteristics. While we performed multivariate and sensitivity analyses to strengthen causality, unmeasured confounders may persist. Third, the sample size, though substantial, may limit the power for certain subgroup analyses. Fourth, the TKI-Mono group was relatively small, reflecting a clinical trend towards incorporating radiotherapy for BM management. Finally, the choice of radiotherapy technique (SRS vs. HA-WBRT) was at the treating physicians’ discretion, although this reflects contemporary practice.

## 5 Conclusion

This study provides compelling evidence that for patients with EGFR-mutant NSCLC and synchronous BMs initiating first-line third-generation TKI therapy, an early combined strategy incorporating upfront brain radiotherapy is associated with significantly improved intracranial control and overall survival compared to a deferred salvage approach, without a significant penalty in treatment-related toxicity. The benefits appear most pronounced in patients with oligometastatic brain disease and possibly those with exon 19 deletions. These results argue against a universal strategy of deferring radiotherapy and instead support a more proactive, integrated approach for selected patients. Prospective randomized trials, such as the ongoing studies addressing this precise question, are urgently needed to validate these findings and establish definitive practice guidelines. Until then, our data suggest that upfront radiotherapy should be strongly considered within a multidisciplinary treatment plan for this patient population.

## Data Availability

The original contributions presented in the study are included in the article/**Supplementary Material**. Further inquiries can be directed to the corresponding author.

## Supplementary material

Supplementary Figure 1 Kaplan-Meier curves for intracranial progression-free survival (iPFS) comparing the Early Combined Therapy (ECT), Salvage Radiotherapy (SRT), and TKI Monotherapy (TKI-Mono) groups in the subgroup of patients with 1–3 brain metastases.

Supplementary Figure 2 Kaplan-Meier curves for overall survival (OS) comparing the Early Combined Therapy (ECT), Salvage Radiotherapy (SRT), and TKI Monotherapy (TKI-Mono) groups in the subgroup of patients with 1–3 brain metastases.

Supplementary Figure 3 Kaplan-Meier curves for intracranial progression-free survival (iPFS) comparing the Early Combined Therapy (ECT), Salvage Radiotherapy (SRT), and TKI Monotherapy (TKI-Mono) groups in the subgroup of patients with >3 brain metastases.

Supplementary Figure 4 Kaplan-Meier curves for overall survival (OS) comparing the Early Combined Therapy (ECT), Salvage Radiotherapy (SRT), and TKI Monotherapy (TKI-Mono) groups in the subgroup of patients with >3 brain metastases.

Supplementary Figure 5 Kaplan-Meier curves for intracranial progression-free survival (iPFS) comparing the Early Combined Therapy (ECT), Salvage Radiotherapy (SRT), and TKI Monotherapy (TKI-Mono) groups in the subgroup of patients with the largest brain metastasis diameter <1 cm.

Supplementary Figure 6 Kaplan-Meier curves for overall survival (OS) comparing the Early Combined Therapy (ECT), Salvage Radiotherapy (SRT), and TKI Monotherapy (TKI-Mono) groups in the subgroup of patients with the largest brain metastasis diameter <1 cm.

Supplementary Figure 7 Kaplan-Meier curves for intracranial progression-free survival (iPFS) comparing the Early Combined Therapy (ECT), Salvage Radiotherapy (SRT), and TKI Monotherapy (TKI-Mono) groups in the subgroup of patients with the largest brain metastasis diameter ≥1 cm.

Supplementary Figure 8 Kaplan-Meier curves for overall survival (OS) comparing the Early Combined Therapy (ECT), Salvage Radiotherapy (SRT), and TKI Monotherapy (TKI-Mono) groups in the subgroup of patients with the largest brain metastasis diameter ≥1 cm.

Supplementary Figure 9 Kaplan-Meier curves for intracranial progression-free survival (iPFS) comparing patients treated with Stereotactic Radiosurgery/Radiotherapy (SRS/SRT) and those treated with Hippocampal-Avoidance Whole-Brain Radiotherapy (HA-WBRT).

Supplementary Figure 10 Kaplan-Meier curves for overall survival (OS) comparing patients treated with Stereotactic Radiosurgery/Radiotherapy (SRS/SRT) and those treated with Hippocampal-Avoidance Whole-Brain Radiotherapy (HA-WBRT).

Supplementary Figure 11 Kaplan-Meier curves for progression-free survival (PFS) comparing patients treated with Stereotactic Radiosurgery/Radiotherapy (SRS/SRT) and those treated with Hippocampal-Avoidance Whole-Brain Radiotherapy (HA-WBRT).
