# Supplementary figures and images for: Early brain radiotherapy combined with third-generation EGFR-TKIs improves survival in EGFR-mutant NSCLC with synchronous brain metastases: a multi-center retrospective analysis
Source: Front Oncol. 2026 Feb 12;16:1770066. doi: 10.3389/fonc.2026.1770066 (PMC12935651; doi:10.3389/fonc.2026.1770066)

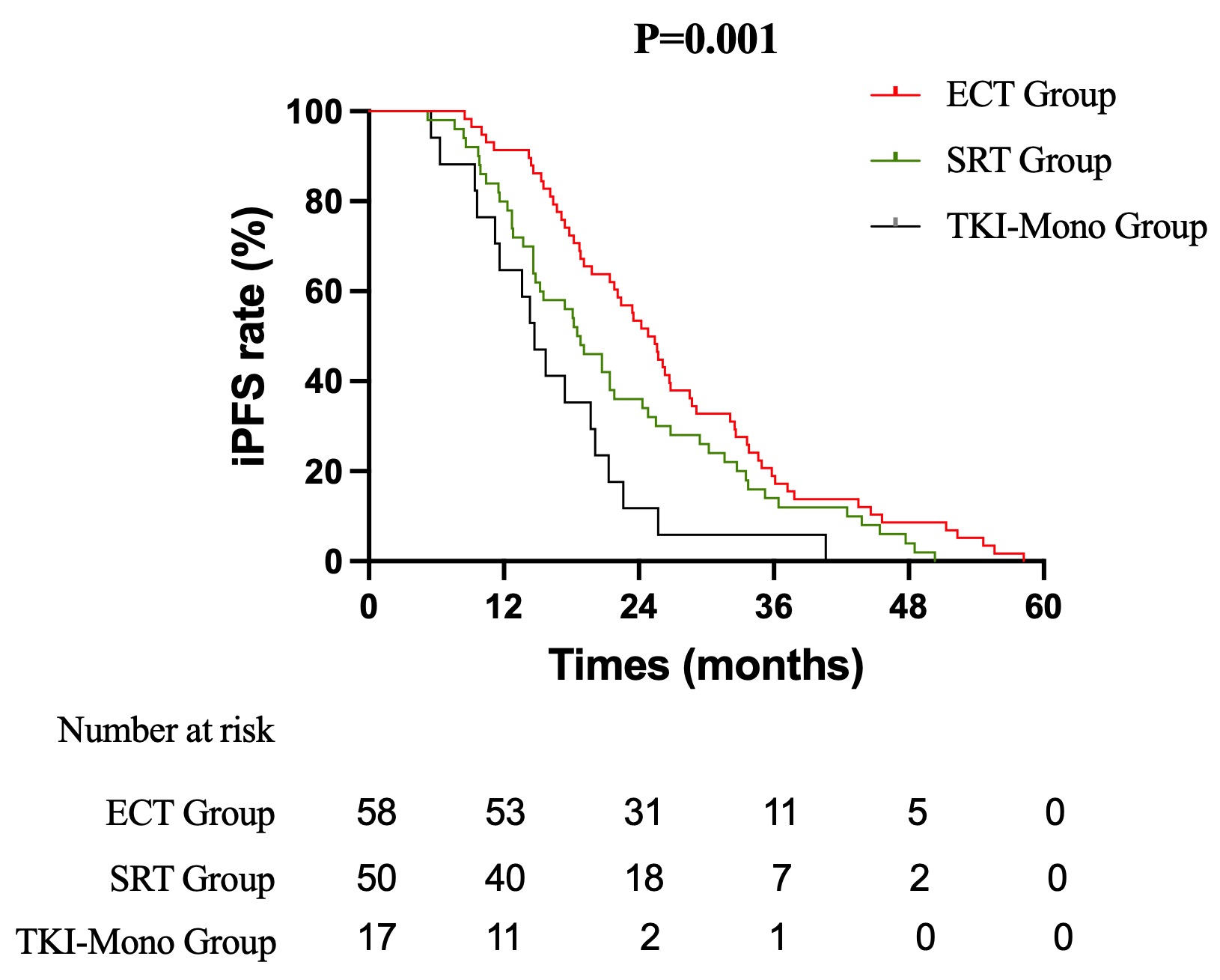

Supplement: Supplementary Figure 1 — Kaplan-Meier curves for intracranial progression-free survival (iPFS) comparing the Early Combined Therapy (ECT), Salvage Radiotherapy (SRT), and TKI Monotherapy (TKI-Mono) groups in the subgroup of patients with 1–3 brain metastases. [file Image1.jpeg]

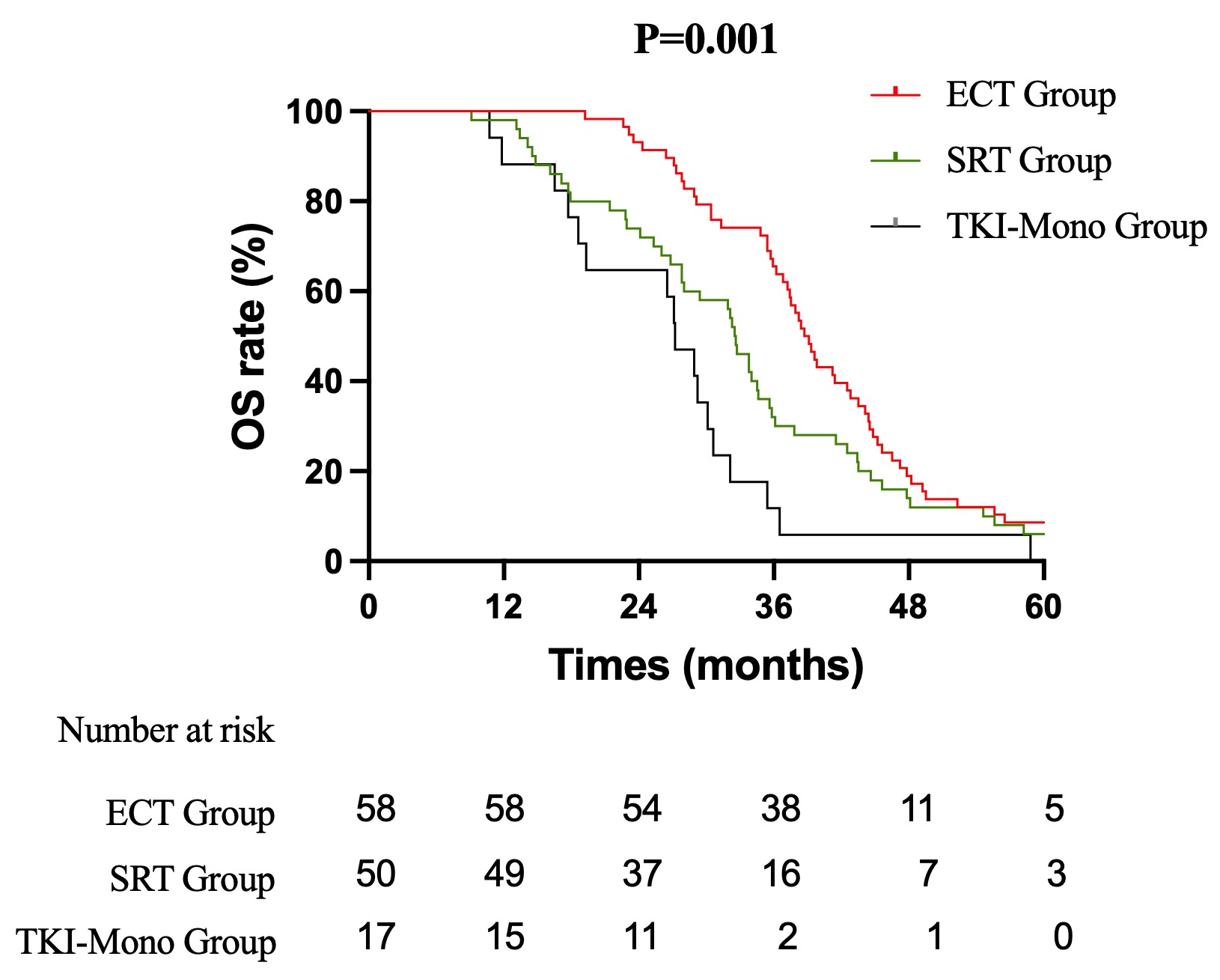

Supplement: Supplementary Figure 2 — Kaplan-Meier curves for overall survival (OS) comparing the Early Combined Therapy (ECT), Salvage Radiotherapy (SRT), and TKI Monotherapy (TKI-Mono) groups in the subgroup of patients with 1–3 brain metastases. [file Image2.jpeg]

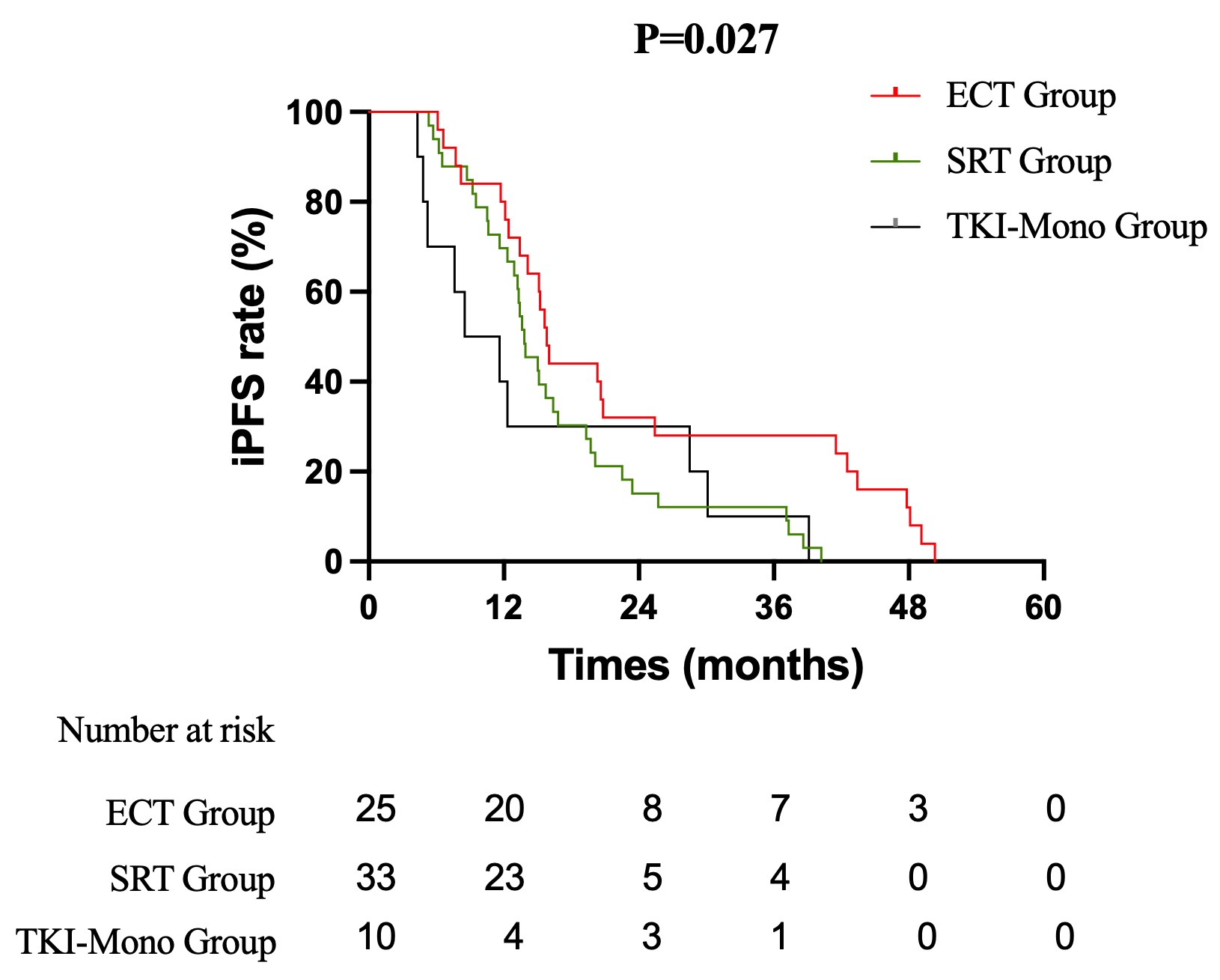

Supplement: Supplementary Figure 3 — Kaplan-Meier curves for intracranial progression-free survival (iPFS) comparing the Early Combined Therapy (ECT), Salvage Radiotherapy (SRT), and TKI Monotherapy (TKI-Mono) groups in the subgroup of patients with >3 brain metastases. [file Image3.jpeg]

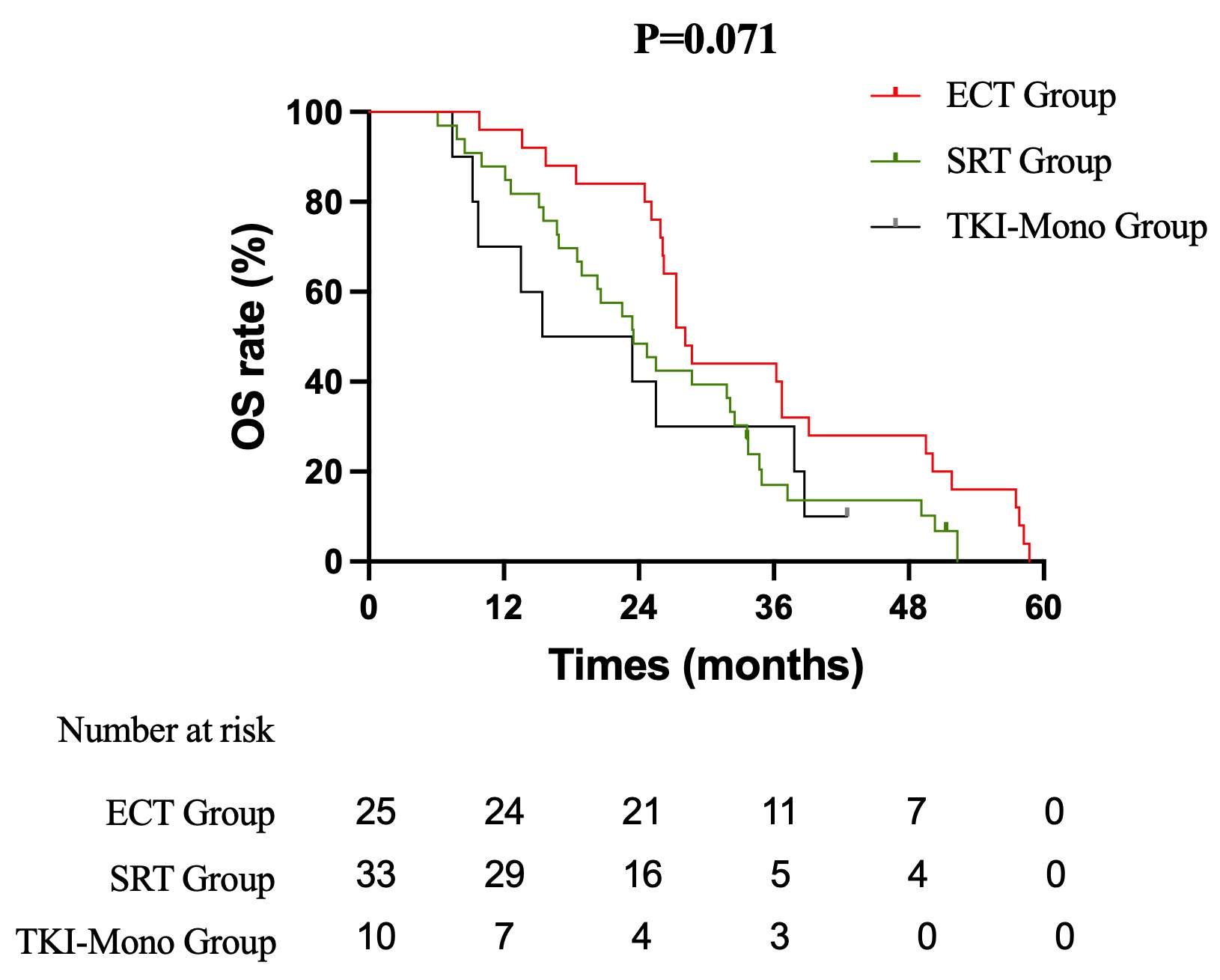

Supplement: Supplementary Figure 4 — Kaplan-Meier curves for overall survival (OS) comparing the Early Combined Therapy (ECT), Salvage Radiotherapy (SRT), and TKI Monotherapy (TKI-Mono) groups in the subgroup of patients with >3 brain metastases. [file Image4.jpeg]

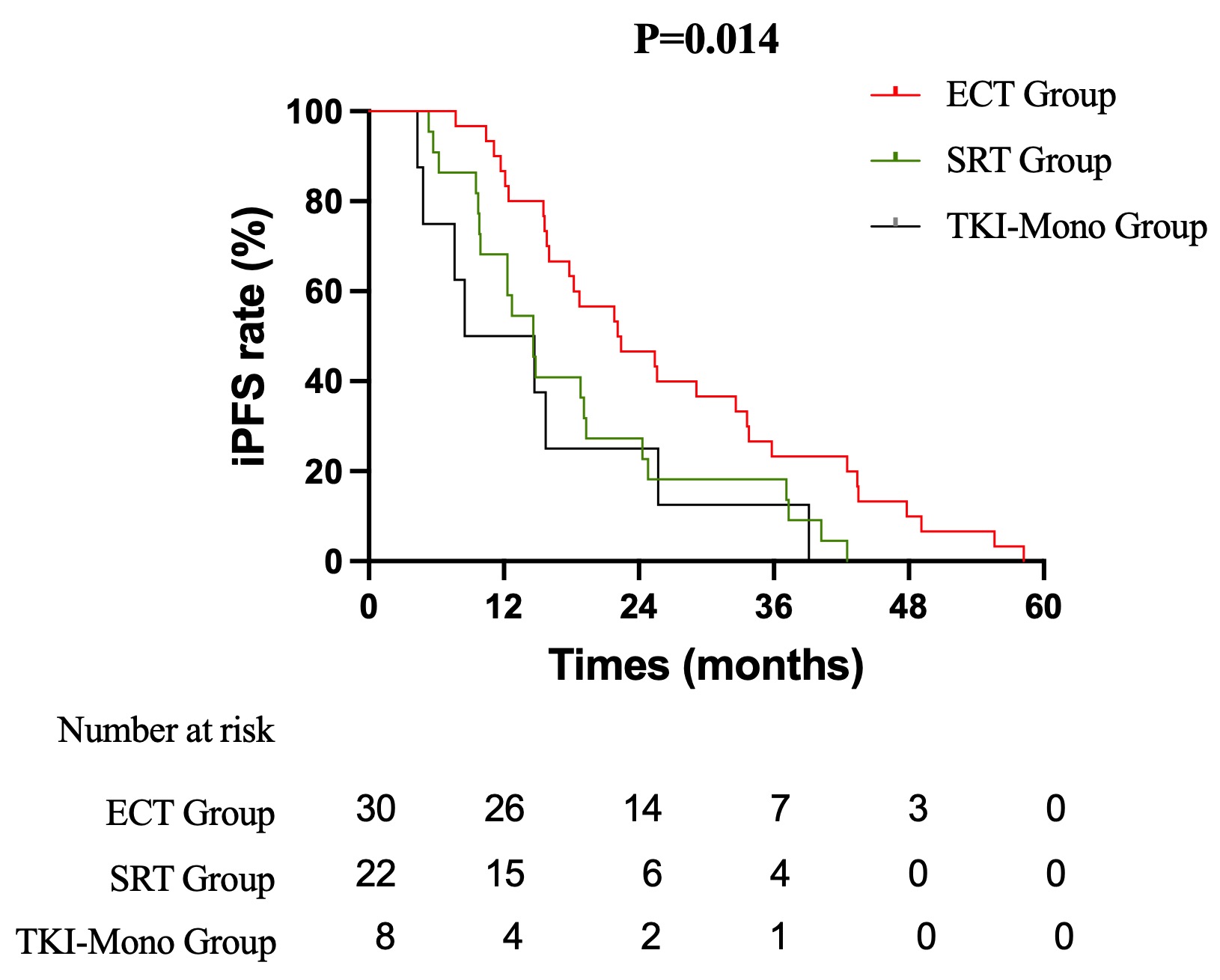

Supplement: Supplementary Figure 5 — Kaplan-Meier curves for intracranial progression-free survival (iPFS) comparing the Early Combined Therapy (ECT), Salvage Radiotherapy (SRT), and TKI Monotherapy (TKI-Mono) groups in the subgroup of patients with the largest brain metastasis diameter <1 cm. [file Image5.jpeg]

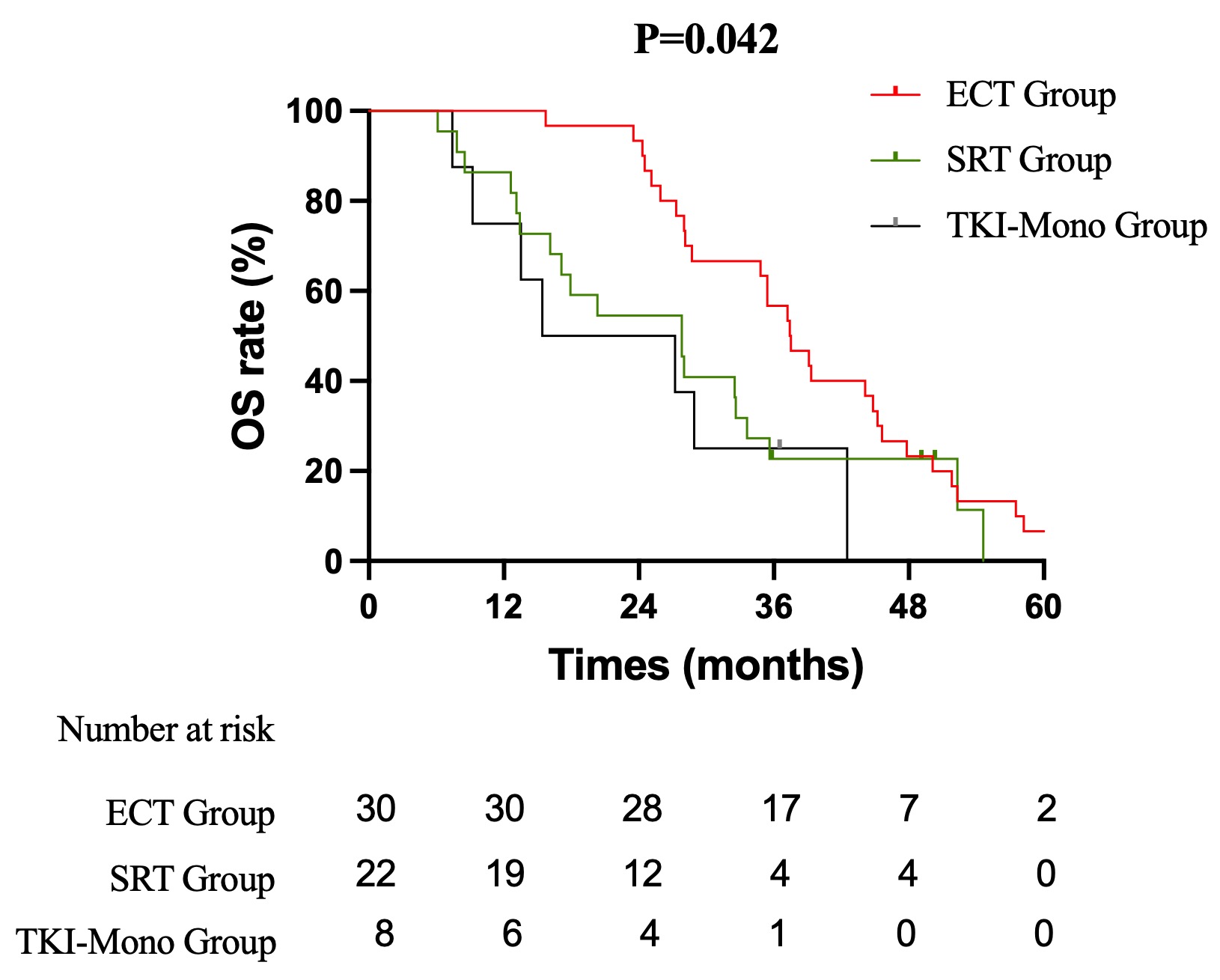

Supplement: Supplementary Figure 6 — Kaplan-Meier curves for overall survival (OS) comparing the Early Combined Therapy (ECT), Salvage Radiotherapy (SRT), and TKI Monotherapy (TKI-Mono) groups in the subgroup of patients with the largest brain metastasis diameter <1 cm. [file Image6.jpeg]

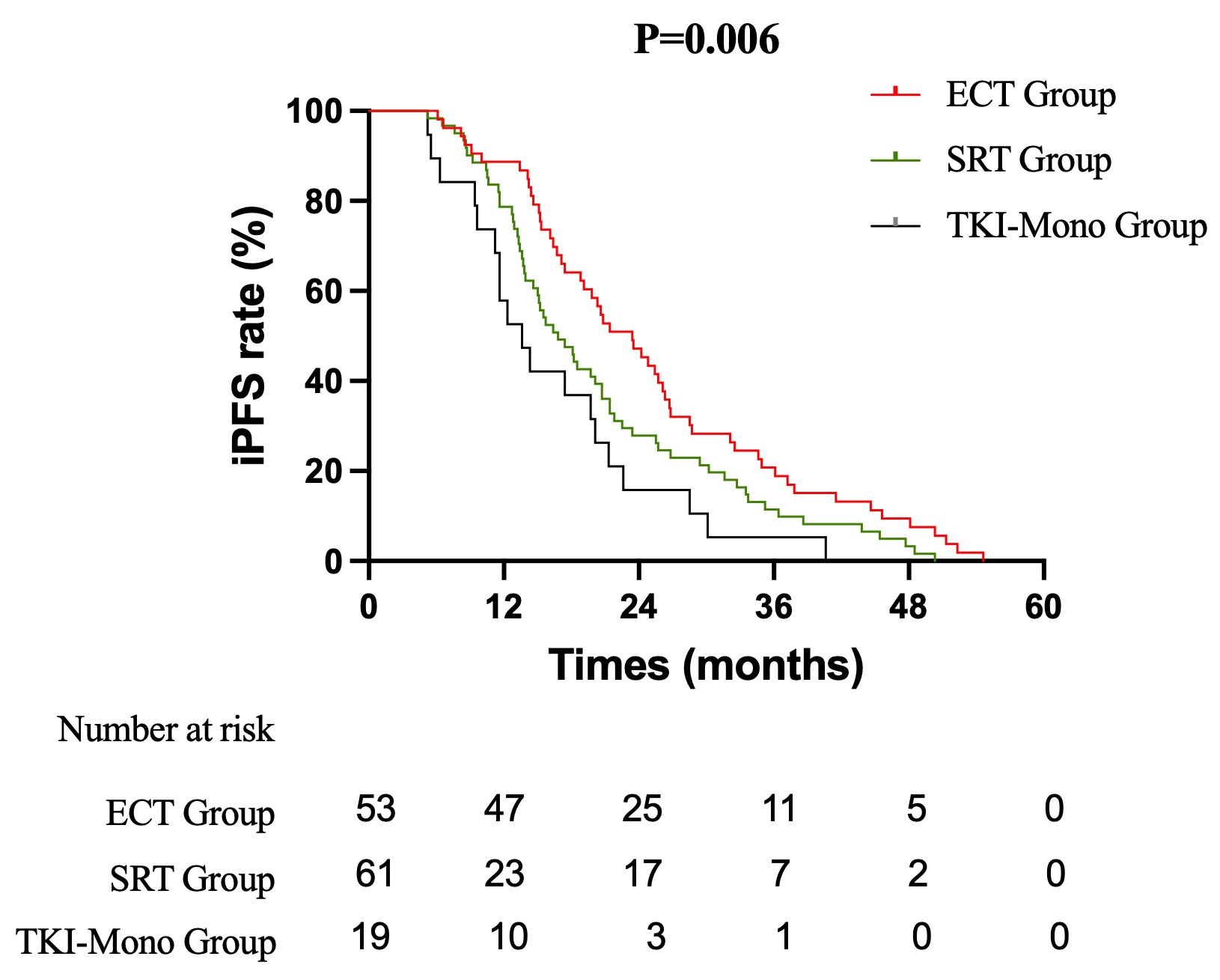

Supplement: Supplementary Figure 7 — Kaplan-Meier curves for intracranial progression-free survival (iPFS) comparing the Early Combined Therapy (ECT), Salvage Radiotherapy (SRT), and TKI Monotherapy (TKI-Mono) groups in the subgroup of patients with the largest brain metastasis diameter ≥1 cm. [file Image7.jpeg]

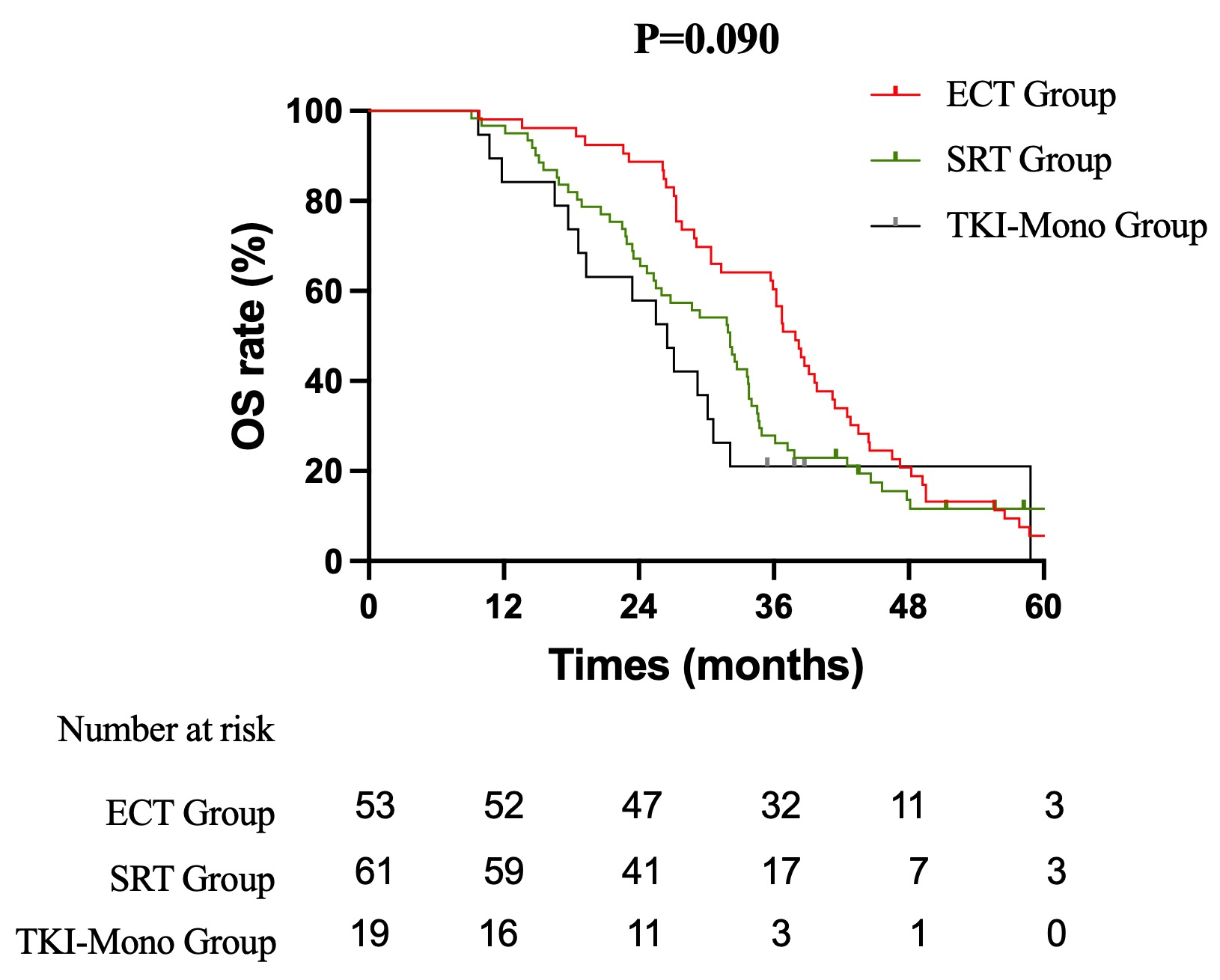

Supplement: Supplementary Figure 8 — Kaplan-Meier curves for overall survival (OS) comparing the Early Combined Therapy (ECT), Salvage Radiotherapy (SRT), and TKI Monotherapy (TKI-Mono) groups in the subgroup of patients with the largest brain metastasis diameter ≥1 cm. [file Image8.jpeg]

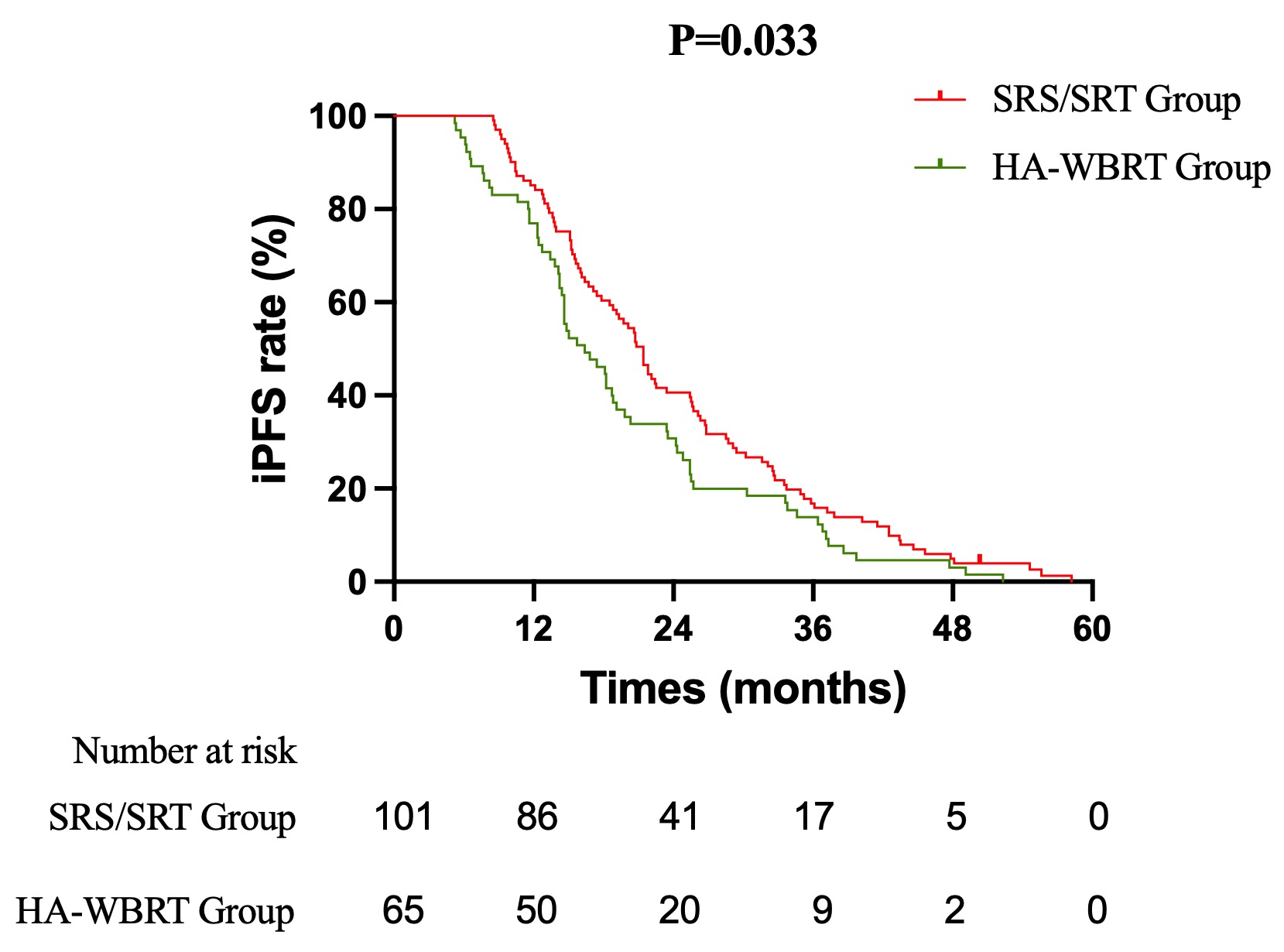

Supplement: Supplementary Figure 9 — Kaplan-Meier curves for intracranial progression-free survival (iPFS) comparing patients treated with Stereotactic Radiosurgery/Radiotherapy (SRS/SRT) and those treated with Hippocampal-Avoidance Whole-Brain Radiotherapy (HA-WBRT). [file Image9.jpeg]

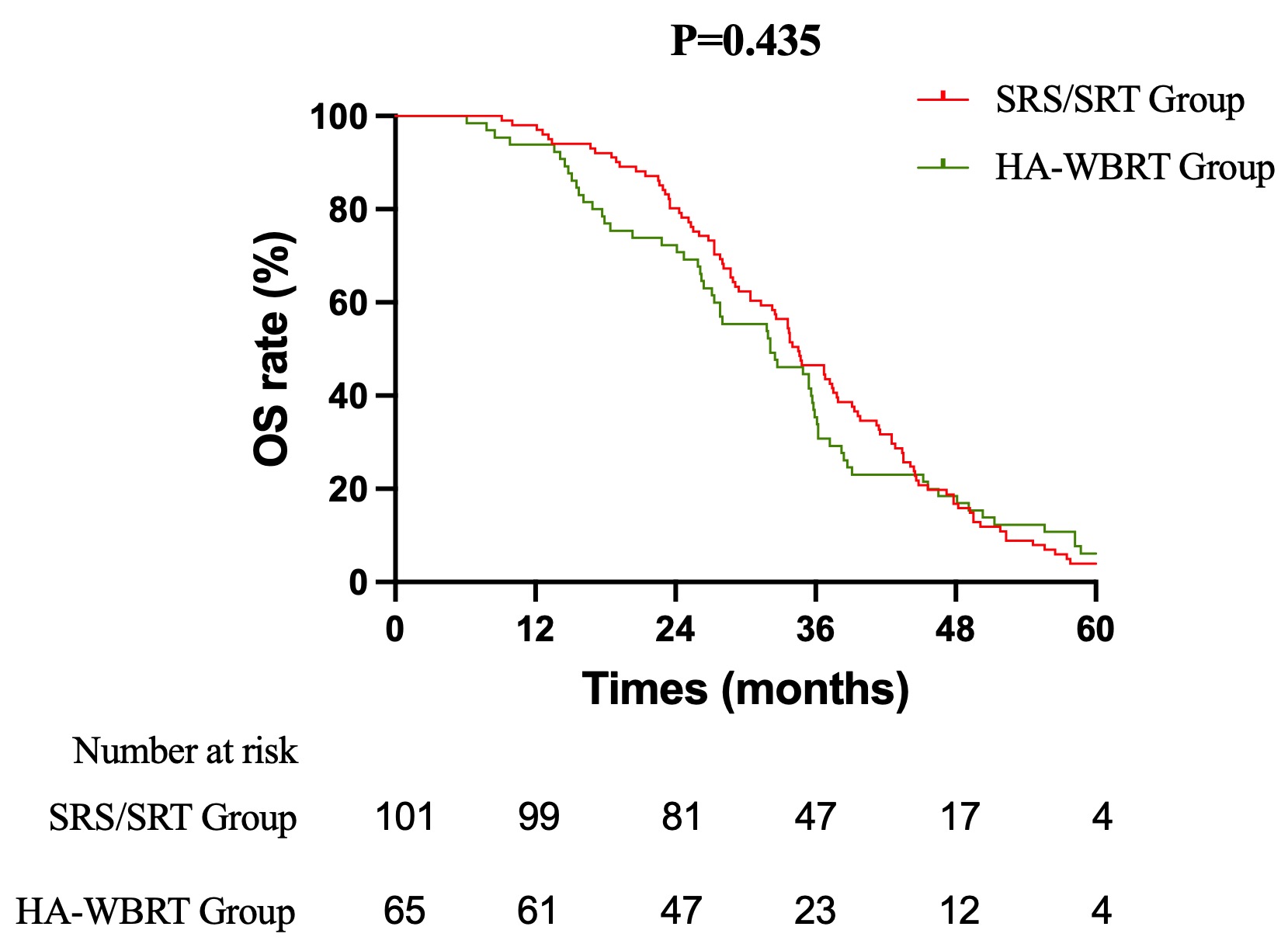

Supplement: Supplementary Figure 10 — Kaplan-Meier curves for overall survival (OS) comparing patients treated with Stereotactic Radiosurgery/Radiotherapy (SRS/SRT) and those treated with Hippocampal-Avoidance Whole-Brain Radiotherapy (HA-WBRT). [file Image10.jpeg]

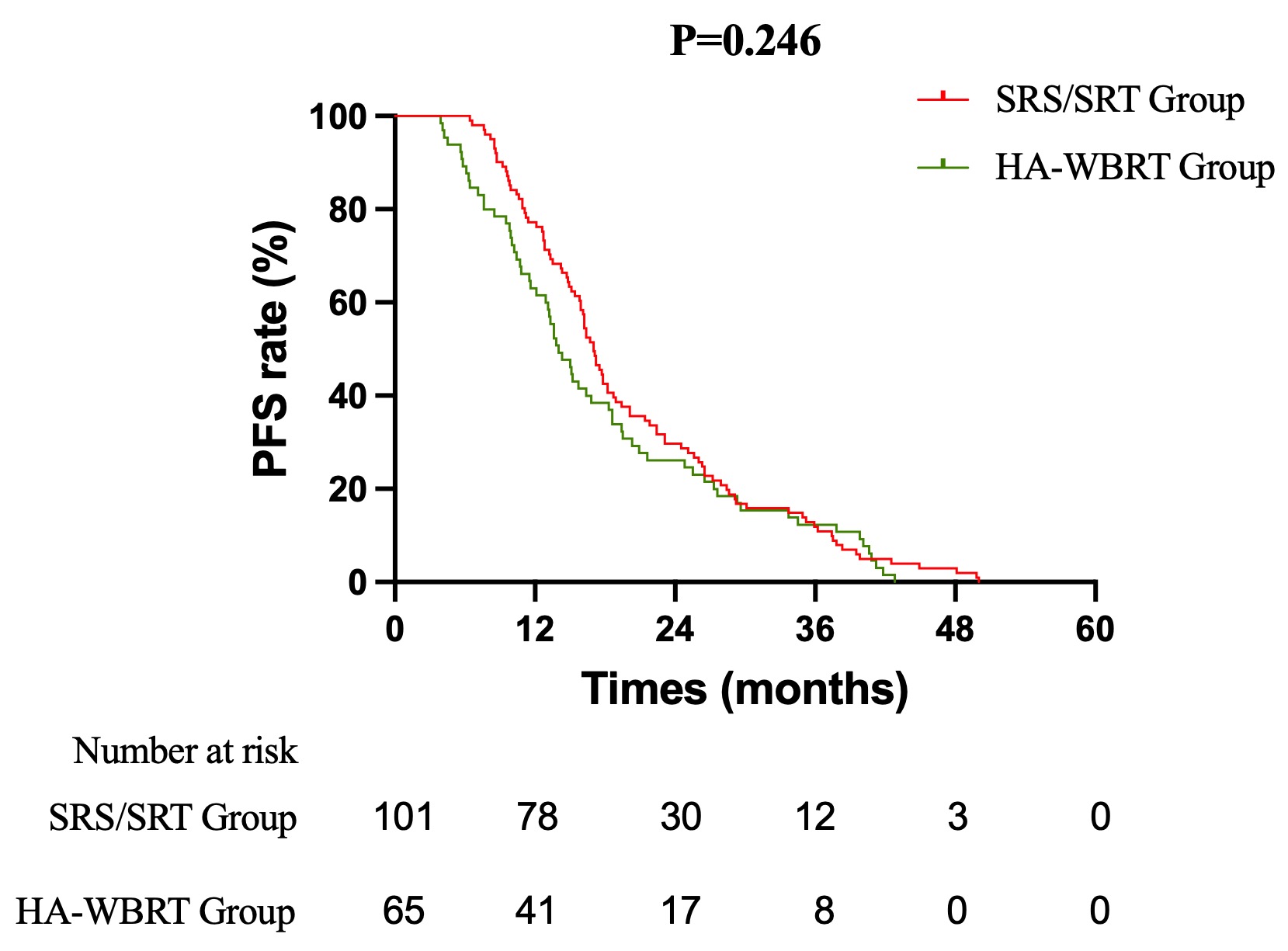

Supplement: Supplementary Figure 11 — Kaplan-Meier curves for progression-free survival (PFS) comparing patients treated with Stereotactic Radiosurgery/Radiotherapy (SRS/SRT) and those treated with Hippocampal-Avoidance Whole-Brain Radiotherapy (HA-WBRT). [file Image11.jpeg]
