# Supplementary material for: Early brain radiotherapy combined with third-generation EGFR-TKIs improves survival in EGFR-mutant NSCLC with synchronous brain metastases: a multi-center retrospective analysis
Source: Front Oncol. 2026 Feb 12;16:1770066. doi: 10.3389/fonc.2026.1770066 (PMC12935651; doi:10.3389/fonc.2026.1770066)
Supplement: Supplementary file 12 [file Table1.docx]

****Supplementary Table 1. Detailed Radiotherapy Parameters for the ECT and SRT Groups.****

| **Parameter** | **ECT Group (n=83)** | **SRT Group (n=83)** | **P value** |
| --- | --- | --- | --- |
| **Radiation Technique, n (%)** |  |  | 0.080 |
| SRS/SRT | 56 (67.5) | 45 (54.2) |  |
| HA-WBRT | 27 (32.5) | 38 (45.8) |  |
| **SRS/SRT Specific Parameters** | **(n=56)** | **(n=45)** |  |
| Prescription Dose, median Gy (range) | 35 (30 - 45) | 35 (30 - 45) | 0.850 |
| Number of Fractions, median (range) | 5 (5 - 10) | 5 (5 - 10) | 0.920 |
| BED10, median Gy (range) | 59.5 (48.0 - 85.5) | 59.5 (48.0 - 85.5) | 0.824 |
| Prescription Isodose Line, median % (range) | 80 (70 - 90) | 80 (70 - 90) | 0.782 |
| CI, median (range) | 1.05 (0.95 - 1.25) | 1.05 (0.95 - 1.25) | 0.915 |
| **HA-WBRT with SIB Specific Parameters** | **(n=27)** | **(n=38)** |  |
| Whole-Brain Prescription Dose | 30 Gy / 10 fractions | 30 Gy / 10 fractions | N/A |
| Boost Dose per Metastasis, median Gy (range) | 15 (10 - 20) | 15 (10 - 20) | 0.780 |
| **Brain Metastasis Location, n (%)** |  |  | 0.583 |
| Supratentorial Only | 65 (78.3) | 62 (74.7) |  |
| With Infratentorial Involvement | 18 (21.7) | 21 (25.3) |  |
| **Organs-at-Risk (OAR) Dose Constraints** |  |  |  |
| Hippocampus: Maximum Dose (Dmax) | < 16 Gy | < 16 Gy | N/A |
| Hippocampus: Mean Dose (Dmean) Goal | ≤ 10 Gy | ≤ 10 Gy | N/A |
| Brainstem: Maximum Dose (Dmax) | < 54 Gy | < 54 Gy | N/A |
| Optic Nerves/Chiasm: Maximum Dose (Dmax) | < 54 Gy | < 54 Gy | N/A |
| Lens: Maximum Dose (Dmax) | < 7 Gy | < 7 Gy | N/A |
| Pituitary Gland: Mean Dose (Dmean) | < 45 Gy | < 45 Gy | N/A |

**Abbreviations:** ECT: Early Combined Therapy; SRT: Salvage Radiotherapy; HA-WBRT, hippocampal-avoidance whole-brain radiotherapy; SIB, simultaneous integrated boost; BED10, biologically effective dose assuming an α/β ratio of 10 Gy for tumor; CI, conformity index.
**Note:** BED10 = nd × (1 + d/(α/β)), where n=number of fractions, d=dose per fraction. α/β was assumed to be 10 Gy for tumor.
